# Integrative analysis of mutational and transcriptional profiles reveals driver mutations of metastatic breast cancers

**DOI:** 10.1038/celldisc.2016.25

**Published:** 2016-08-30

**Authors:** Ji-Hyun Lee, Xing-Ming Zhao, Ina Yoon, Jin Young Lee, Nam Hoon Kwon, Yin-Ying Wang, Kyung-Min Lee, Min-Joo Lee, Jisun Kim, Hyeong-Gon Moon, Yongho In, Jin-Kao Hao, Kyung-Mii Park, Dong-Young Noh, Wonshik Han, Sunghoon Kim

**Affiliations:** 1 Medicinal Bioconvergence Research Center, College of Pharmacy, Seoul National University, Seoul, Republic of Korea; 2 Research Institute of Pharmaceutical Sciences, College of Pharmacy, Seoul National University, Seoul, Republic of Korea; 3 Department of Computer Science and Technology, Tongji University, Shanghai, China; 4 Department of Surgery, Seoul National University College of Medicine, Seoul, Republic of Korea; 5 LERIA, University of Angers, Angers, France; 6 Cancer Research Institute, Seoul National University, Seoul, Republic of Korea; 7 Department of Molecular Medicine and Biopharmaceutical Sciences, Seoul National University, Seoul, Republic of Korea

**Keywords:** driver mutations, exome sequencing, integrative analysis, metastatic breast cancer, RNA sequencing

## Abstract

Despite the explosion in the numbers of cancer genomic studies, metastasis is still the major cause of cancer mortality. In breast cancer, approximately one-fifth of metastatic patients survive 5 years. Therefore, detecting the patients at a high risk of developing distant metastasis at first diagnosis is critical for effective treatment strategy. We hereby present a novel systems biology approach to identify driver mutations escalating the risk of metastasis based on both exome and RNA sequencing of our collected 78 normal-paired breast cancers. Unlike driver mutations occurring commonly in cancers as reported in the literature, the mutations detected here are relatively rare mutations occurring in less than half metastatic samples. By supposing that the driver mutations should affect the metastasis gene signatures, we develop a novel computational pipeline to identify the driver mutations that affect transcription factors regulating metastasis gene signatures. We identify driver mutations in ADPGK, NUP93, PCGF6, PKP2 and SLC22A5, which are verified to enhance cancer cell migration and prompt metastasis with *in vitro* experiments. The discovered somatic mutations may be helpful for identifying patients who are likely to develop distant metastasis.

## Introduction

Despite the advances in early detection and adjuvant treatment, breast cancer is still the leading cause of cancer mortality in women, where most of such patients die from metastatic disease. Approximately 4–6% of breast cancers are metastatic at diagnosis. The vast majority of metastatic breast cancer is incurable, and approximately one-fifth will survive for 5 years [1]. As the survival rate of metastatic breast cancer is extremely low, it is important to detect patients with high risk for distant metastasis (HRM) at first diagnosis and design effective treatment strategies. In the past decade, large-scale cancer genome studies and comprehensive analysis have been performed, and a number of somatically acquired changes have been observed in cancer [2–4]. It is well established that each tumor is unique and typically exhibits a large number of somatic mutations. However, only few mutations have ‘driver’ roles in tumorigenesis and metastasis, whereas the others are ‘passengers’ that have simply been accumulated during the growth of the cancer and do not have any functional effect on cancer development [5]. The biggest challenge in cancer research is to detect true driver mutations and to distinguish them from the random passenger mutations [6].

To identify the driver mutations that are responsible for the development and aggressiveness of cancer, many large-scale genomic analyses have been carried out based on the assumption that the driver mutations should recurrently occur in cancers. Unfortunately, as the size of sample increases, the list of identified cancer-associated genes grows implausibly. In other words, many driver mutations actually occur with relatively low frequencies (for example, in <10% of samples). For example, a recent genomic study of breast cancer reported that only 8 of >40 driver genes were mutated in at least 10% of breast cancers [7]. In another much larger-scale genome-wide sequencing studies of 3 284 tumors, it was estimated that only 125 genes that contain driver mutations have been discovered [8]. Therefore, more efficient and sophisticated computational approaches are highly demanded to identify those rare driver mutations.

In this study, we propose a novel systems biology approach to identify the key driver somatic mutations escalating the risk of distant metastasis in breast cancers. In our computational approach, we suppose that the drive mutations should occur exclusively [9, 10] and transduce their signals to certain transcription factors (TFs) that regulate those genes expressed differentially in metastasis, where the differentially expressed gene (DEG) are regarded as metastatic signatures [11, 12]. With these assumptions, we first built a mathematic model to pick up candidate drive mutations by taking into account the exclusiveness between driver mutations, and then the pathways linking mutations to metastatic signatures were identified with a network flow model and the mutations involved are regarded as potential driver mutations. By applying to the exome and RNA sequencing data of 78 normal-paired breast cancer samples we collected from 1998 to 2008, our approach identifies five driver mutations in ADPGK, NUP93, PCGF6, PKP2 and SLC22A5 genes. With *in vitro* experiments, we verify these mutations to be able to enhance cell migration and prompt metastasis, implying the important roles of these mutations in breast cancer metastasis.

## Results

**Figure 1:**
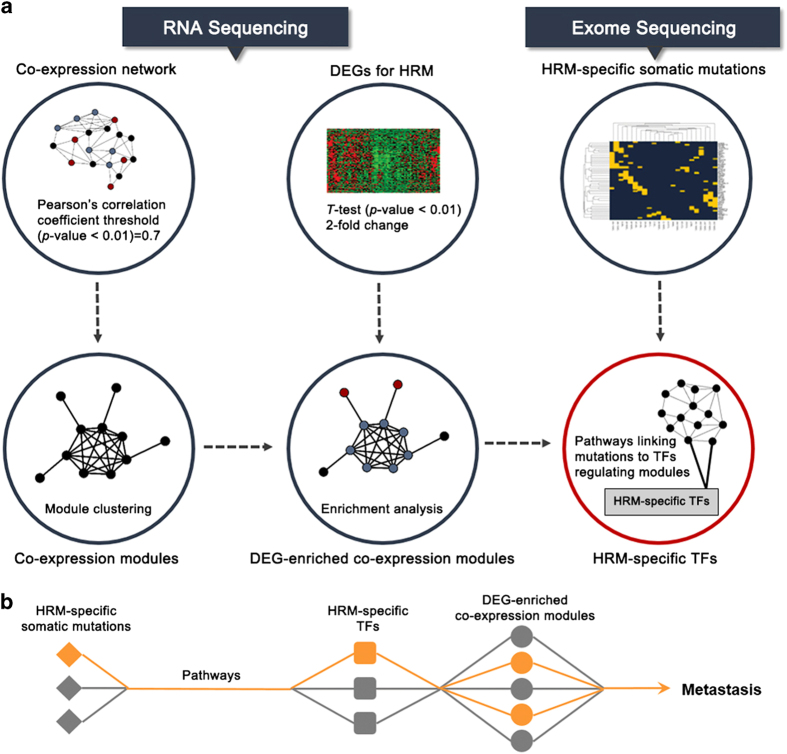
Integrative analysis pipeline to predict signaling pathways affected by HRM (high risk for distant metastasis)-specific mutations. (**a**) Integrative analysis workflow using RNA and Exome sequencing data. Co-expression modules were generated using FPKM (fragments per kilobase of gene model per million mapped reads) values from RNA sequencing and co-expression network analysis. The modules that differentially expressed genes (DEGs) and transcription factor (TF) targets enriched (*P*-value<0.01) were selected as DEG-enriched co-expression modules. (**b**) The conceptual flow diagram of the analysis.

To identify the key somatic mutations responsible for the risk of distant metastasis in breast cancers, we proposed a novel systems biology pipeline as shown in Figure 1. First, a small set of somatic mutations that occur exclusively in metastatic samples were picked up from exome sequencing data with a mathematic model; second, the network signatures of breast cancer metastasis (denoted as HRM) were identified from a gene co-expression network based on our RNA sequencing data; third, the TFs that regulate the HRM-specific network signatures were detected; fourth, the pathways linking mutations to TFs were identified with a network flow model, and the mutations involved in the pathways were regarded as potential driver mutations and went through experimental validations. The details will be addressed in the following parts.

**Figure 2:**
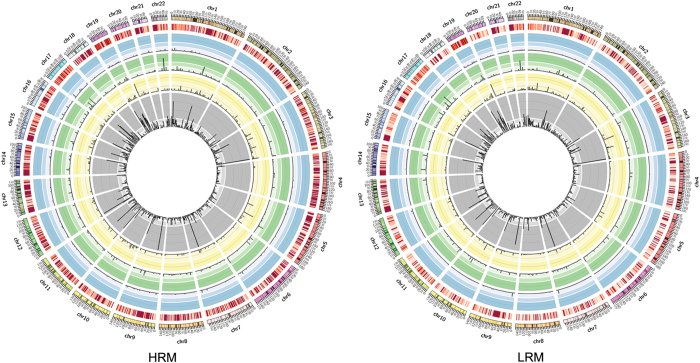
Mutational landscape of breast cancer. Distribution of somatic mutations in 22 HRM (high risk for distant metastasis) breast cancer patients (left) and 56 LRM (low risk for distant metastasis) breast cancer patients (right). In the inner gray circle, histogram denotes the average frequency of missense mutations; in the second yellow circle, histogram denotes the average frequency of frameshift InDels; in the third green circle, histogram denotes the average frequency of in-frame InDels; in the fourth blue circle, histogram denotes the average frequency of nonsense mutations; and in the fifth red circle, each block denotes the average frequency of somatic mutations, and the color gradually changes from light red to dark red as the frequency is increased.

With exome sequencing, the total of 3 685 and 8 043 somatic mutations were identified in HRM and LRM (low risk for distant metastasis) samples, respectively (Table 1, Supplementary Table S1, Supplementary Table S2). The mutational landscape of two groups are displayed in Figure 2 from which no significant difference can be detected between the two groups. The HRM group (average=226.2, ranging from 113 to 485) showed slightly higher somatic mutation frequency than the LRM group (average=214.9, ranging from 81 to 626) (*P*-value=0.33, one-sided *t*-test; Supplementary Figure S1A). Furthermore, there was no dramatic difference in the mutation-type comparison between two groups (Supplementary Figure S1B). The significant positive correlation was observed between histological grade and number of somatic mutations (*P*-value=0.038, Wilcoxon rank-sum test). We compared the prevalence of mutations in our data sets (*n*=78, BC) with their prevalence in The Cancer Genome Atlas (TCGA) invasive breast cancer (*n*=507) (Supplementary Figure S2). The prevalence of mutations in BRCA2, CDH1, FGFR2, GATA3, PTEN and TP53 was comparable in all sets. Although the importance of CDH1 inactivation for tumor metastasis has been well demonstrated in several studies [13, 14], its mutation frequency was not significantly high and was <10% across all data sets. In contrast, ARID1A (TCGA=2%, BC=13%), NOTCH2 (TCGA=1%, BC=18%) and USH2A (TCGA=5%, BC=26%) mutations were detected at much higher rates in our samples than in those reported by TCGA. Frequent somatic mutations in ARID1A, a tumor suppressor, have been reported in a variety of human cancers [15–17]. In our BC cases, p.Q1333_Q1334del mutation was detected in six cases. Based on the report in which the cells expressing the p.Q1134_R1335dupQ mutation exhibited attenuated growth inhibition, it can be carefully suggested that ARID1A p.Q1333_Q1334del mutation can result in loss of ability to suppress cellular proliferation [18]. Another difference was observed in NOTCH2. The prevalence of mutations in NOTCH2 was significantly higher in our BC data sets (5 cases among 507 TCGA cases, 14 cases among 78 BC cases). And nearly 80% (*n*=12) of NOTCH2 mutations in BC data sets was a novel p.R5_P6del frameshift mutation. As NOTCH can have tumor-promoting and -suppressive roles depending on the cellular context, more deeper functional study is required to reveal its role in breast cancer [19]. On the other hand, some mutations were rarely detected in our samples. For example, ESR1 mutations have been reported in 32% of estrogen receptor-positive metastatic breast cancers [20] and in 0.4% of the cases in TCGA, while no somatic mutations were detected for ESR1 in our 10 estrogen receptor-positive HRM cases.

### HRM-specific somatic mutations

**Figure 3:**
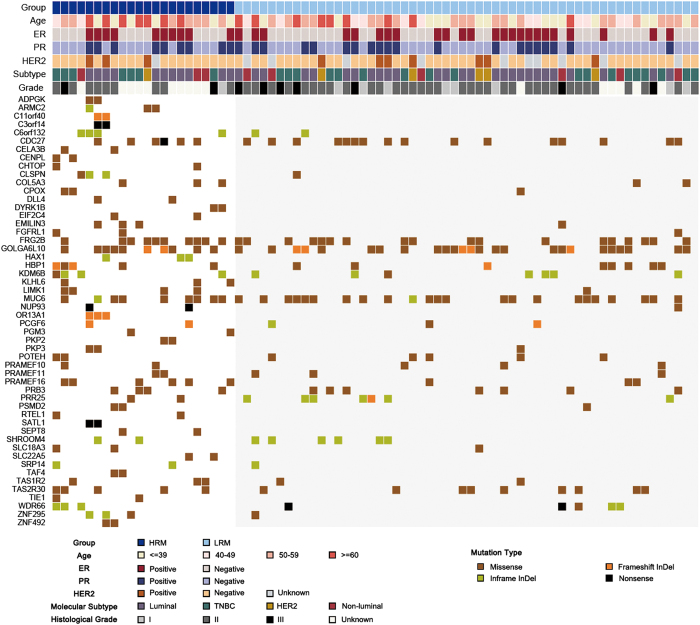
Overview of the mutation status of genes containing HRM (high risk for distant metastasis)-specific mutations. Mutational status is shown for HRM (*n*=22) and LRM (low risk for distant metastasis; *n*=56) groups.

As the type and the position of mutations are critical factors, we focused on identifying HRM-specific somatic mutations rather than recurrently mutated genes. As a result, we identified 53 somatic mutations that were specifically found in the HRM group but were absent in the LRM group (Figure 3, Supplementary Table S3), where the mutations can be found in >9% of HRM patients but not found in LRM patients. Generally, the genes with HRM-specific mutations may also contain other types of mutations at different positions. But there were some unique genes (such as ADPGK, C11orf40, C3orf14, CELA3B, CENPL, CHTOP, DYRK1B, EIF2C4, HAX1, KLHL6, OR13A1, PGM3, PKP2, RTEL1, SATL1, SEPT8, SLC22A5, TAF4, TIE1, ZNF492) that only have one kind of mutation in the HRM group and no other mutations at all in the LRM group. The HRM-specific mutations were distributed evenly across the samples and no mutations occur in >50% of the samples (Supplementary Figure S3). Furthermore, surprisingly none of those mutant genes were found to be expressed differentially in HRM samples. Without considering the functional contexts of mutations, it is impossible to determine which one is a driver here. Therefore, we have developed a new integrated approach in order to distinguish driver mutations from passenger mutations.

### Identification of potential driver mutations in HRM

Recently, it has been found that mutations in cancer tend to be mutually exclusive [9, 10]. With this in mind, we proposed a simple mathematic model to identify the minimum set of mutations that can cover all the patients and assumed that these mutations are more likely driver mutations. As a result, 18 such mutations were detected by our mathematic model, and no significant differential expression patterns were detected for these genes between the HRM and LRM groups. The results on both mutation distribution across HRM samples and expression of mutant genes indicate that the HRM-specific mutations are relatively rare mutations and they are not directly responsible for the DEGs, which are generally regarded as gene signatures of cancer.

Based on the above findings, we hypothesized that genetic mutations responsible for the metastasis will transfer their signals to some TFs through certain signaling pathways, and the TFs will perturb the downstream processes that result in DEGs (Figure 1b). With this hypothesis, we have developed a novel approach to identify the pathways that link the above 18 mutations to TFs regulating DEGs. In addition, except for the 18 mutations, we also considered the genes that have mutations occurring only in HRM samples. To identify the TFs regulating DEGs, we assumed that genes with similar expression profiles were regulated by common TF(s). We constructed a co-expression network based on the RNA sequencing data of HRM samples, where a pair of genes were linked if their Pearson’s correlation coefficient was >0.7 with *P*-value of 0.01. The ClusterONE [21] tool was applied to identify modules from the co-expression network with default parameters. The genes belonging to the same modules were regarded to be regulated by the same TF if its target genes were also enriched in the module (*P*-value<0.01) where the TF–gene regulations were extracted from the UCSC Table browser [22]. In this way, the TFs regulating DEGs enriched modules (*P*-value<0.01) can be identified (Supplementary Table S4), and these DEG modules were regarded as HRM-specific network signatures hereafter. With the genetic mutations and HRM-specific network signatures, we detected the pathways linking them with our previously developed network flow model [23, 24] (for details, refer to Meterials and Methods). After overlaying the genes over a protein–protein interaction network obtained from HPRD (release 9), 13 pathways were detected with each for one mutant gene. Note that not all mutations can be linked to TFs owing to the incompleteness of current interactome. With the pathways, the 13 mutant genes were further ranked based on the weights accompanying their pathways (see Meterials and Methods). Supplementary Table S5 shows the ranking of the 13 mutant genes and those selected by the mathematic model were also marked. The detailed pathways can be found in Supplementary Table S6.

Among the 13 mutant genes, there are some genes that have been reported to be involved in metastasis. For example, Delta-like ligand 4 (DLL4), a ligand for the Notch family of receptors, has been found to be able to forecast the prognosis of several human malignancies [25–27]. Recently, DLL4 was found to be overexpressed in axillary lymph node metastasis and was a good biomarker for poor prognosis in breast cancer [28]. As a member of the LIM kinase (LIMK) family widely involved in cell motility and invasion, the overexpression of LIMK1 was found to increase the motility of human breast cancer cell lines [29] and was found to prompt tumor angiogenesis and induce metastasis to the livers and lungs in breast cancer [30]. The inhibition of LIMK1 activity was found to be able to reduce breast cancer growth and invasiveness, indicating the important role of LIMK1 in breast cancer metastasis [31]. HAX1 has been found to have important roles in neoplastic transformation of several types of tumors, including breast cancer [32], and was found to interact with urokinase-type plasminogen activator receptor implicated in tumor growth and metastasis [33]. The TIE1 gene has been found to be downregulated in lymph node-positive canine mammary carcinomas [34], while the anti-HER2 antibody trastuzumab can be used for patients with metastatic breast cancers that overexpress HER2 by stimulating DYRK1B [35]. These findings imply that the five genes are indeed involved in breast cancer metastasis.

Except for the mutant genes, multiple TFs in the 13 pathways have also been reported to be related to breast metastasis. For instance, the TF SP1 was found to regulate oncogenic protein kinase Cε and urokinase-type plasminogen activator receptor that were strongly associated with tumor aggressiveness and malignant transformation and metastasis [36, 37], and SP1 itself was also reported to be involved in the invasion and metastasis of breast cancer [38]. The activity of ELK1 was found to be positively associated with estrogen receptor and related to breast neoplasia [39]. The peroxisome proliferator-activated receptor-gamma was found to have important roles in the development and progression of breast cancer [40], and the peroxisome proliferator-activated receptor-gamma ligands have been found to reduce migration and invasion of breast cancer cells [41, 42]. The loss of transcriptional activity of USF2 in breast cancer cell lines has been reported [43]. The aberrant expression of PAX5 has been found to be associated with metastatic mammary carcinoma [44], and the gene has much higher expression in metastatic lymph node tumors [45]. All these evidences indicate that the TFs affected by the mutant genes have important roles in metastasis, which also proves to some extent that our detected mutations are potential driver mutations.

### Validation of candidate driver mutations

**Figure 4:**
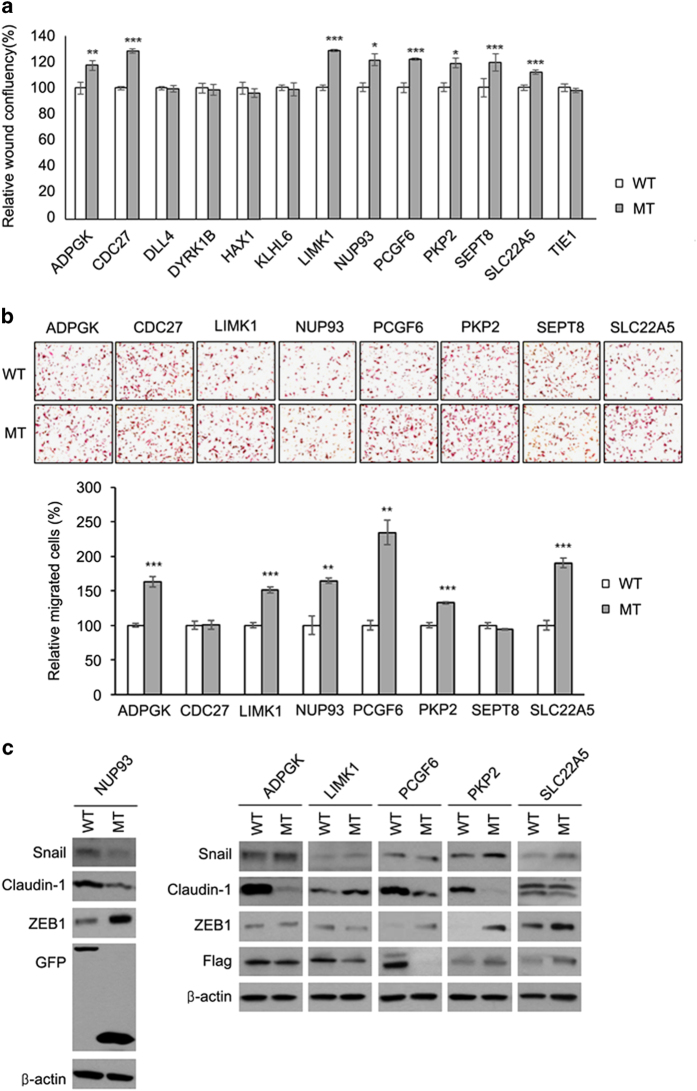
Experimental validation for the effect of selected mutations on metastasis. (**a**) Relative percentage of wound recovery. Scratch wound-healing assay was performed using MDA-MB-231 cells expressing the wild-type (WT) and mutant (MT) proteins of 13 selected genes. The relative recovery of wound region was calculated by cell confluency and is presented as bar graph. ***P<0.001; **P<0.01; and *P<0.05. (**b**) Relative percentage of migratory cells penetrating into the surrounding space. Transwell migration assay was carried out with MDA-MB-231 cells expressing the WT and MT proteins of eight selected genes. The representative staining images of migratory cells (upper) and the relative percentage values (lower) are shown, respectively. ***P<0.001; and **P<0.01. (**c**) EMT markers affected by the WT and MT protein expression. MDA-MB-231 cells were transfected with each plasmid encoding the WT and MT proteins, and the cell lysates were collected and subjected to the sodium dodecyl sulfate–polyacrylamide gel electrophoresis and immunoblotting. The gels have been run under the same experimental conditions.

To validate whether the above 13 mutations that showed high potentials are driver mutations, we performed three *in vitro* experiments. First, we expressed wild-type (WT) or mutant (MT) proteins of the selected genes in MDA-MB-231 cell line and investigated the mutational effect on cell migration via wound-healing assay (Figure 4a, Supplementary Figure S4). Expression of 5 proteins (DLL4, DYRK1B, HAX1, KLHL6 and TIE1) out of the 13 did not show any significant differences between WT and MT. On the other hand, mutations in eight proteins (ADPGK, CDC27, LIMK1, NUP93, PCGF6, PKP2, SEPT8 and SLC22A5) significantly enhanced the cell migration compared with the corresponding WT. Therefore, these eight mutants underwent further validation.

Second, we carried out transwell migration experiments. In wound-healing assay, the migration is calculated by the extent of horizontal movement to the wound region; therefore, it is basically affected by the proliferation rate. To make up for this point and investigate the penetrating ability into the surrounding space, we carried out transwell migration assay on the selected eight genes (Figure 4b). Among them, six MTs significantly increased the cell migration as expected, and PCGF6 MT showed the best migration activity compared with corresponding WT. MTs of CDC27 and SEPT8 did not show significant differences.

**Figure 5:**
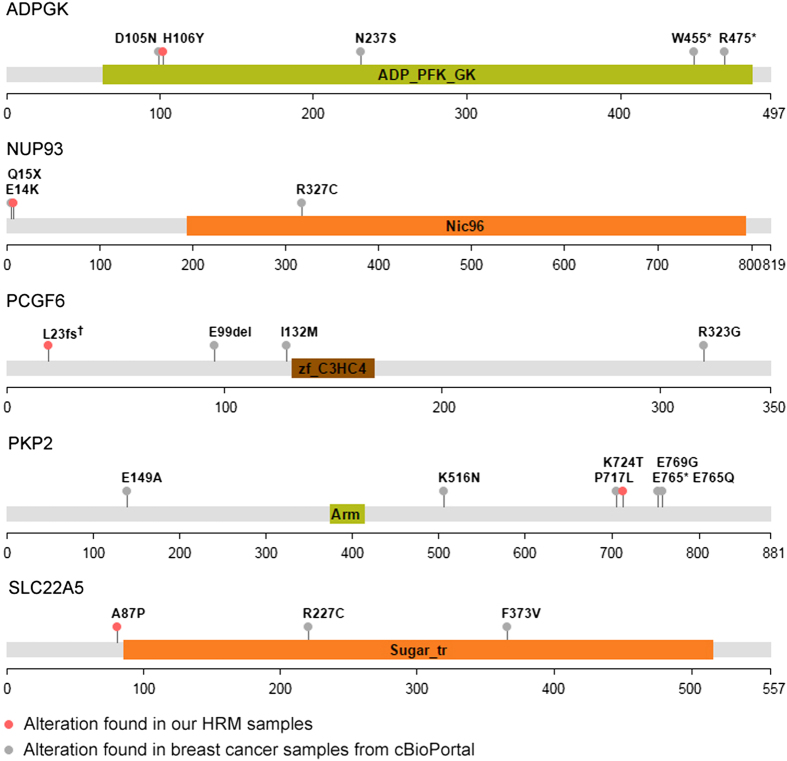
Distributions of protein alterations encoded in validated driver genes escalating the risk of the metastasis. ^†^Found in both cases.

Third, we further examined to find whether these six MTs can affect metastatic signaling pathway. During the metastasis, epithelial-to-mesenchymal transition (EMT) is essential and Snail, Claudin-1 and ZEB1 are well-known markers for metastasis in breast cancer [46–51]. It is known that TF Snail increases the expression of ZEB1, and both Snail and ZEP1 increase EMT. Claudin-1 controls several genes related to the EMT, and knockdown of Claudin-1 is related to recurrence status. We detected the expressional change of Snail, Claudin-1 and ZEB1 in the WT- and MT-expressing MDA-MB-231 cells with regard to the six genes that revealed significant change in the wound recovery and transwell migration (Figure 4c). As shown in Figure 4c, the LIMK1 MT did not show any clear expressional change in these EMT markers tested, but MTs of ADPGK, NUP93, PCGF6 and PKP2 clearly decreased the level of Claudin-1. Levels of ZEB1 are apparently increased by overexpression of the NUP93, PCGF6, PKP2 and SLC22A5 MTs. Mutations in ADPGK, PKP2 and SLC22A5 also enhanced the level of Snail. The mutation in PCGF6 gene induces early stop of translation resulting in the production of undetectably small protein; therefore, we could not confirm the expression of PCGF6 MT via western blotting (Figure 5). All of the results suggest that MTs of ADPGK, PCGF6, PKP2, NUP93 and SLC22A5 can be the driver mutations controlling the cancer metastasis via affecting the EMT pathways where Snail, Claudin-1 or ZEB1 is involved.

## Discussion

We present here a novel computational approach to identify driver mutations escalating metastatic breast cancer based on integrative analysis of mutational and transcriptional profiles. Unlike the traditional large-scale sequencing work, most of the mutations detected here occur in less than half of the samples. Based on the newly developed analysis approach, we discovered five driver mutations in ADPGK, NUP93, PCGF6, PKP2 and SLC22A5 genes, which enhanced migration and induced the protein level changes of EMT markers. PKP2 is an activator of epidermal growth factor receptor, and its overexpression is related to cancer malignancy [52]. As PKP2 is originally an activator for cancer metastasis, the mutation of PKP2 can be a hyperactive variant. Meanwhile, other four genes are not well known yet for their roles and mechanisms in cancer metastasis. High expression of SLC22A5 in breast cancer was reported [53]. NUP93 was identified as one of the top 10 breast cancer drivers, based on the impacts on global gene expression, and the amplification of NUP93-containing chromosome was reported in breast cancer [54, 55]. We further verified these mutations with *in vitro* experiments, and these mutations were found to enhance cell migration and induce protein level changes of the EMT markers, indicating the important roles of these mutations in metastasis. These driver mutations can be considered as prognosis markers of distant metastasis and help design treatment strategies at the time of initial diagnosis. Using our analysis approach, the pathways altered by these driver mutations can be suggested (Supplementary Figure S5). However, more research needs to be undertaken before the association between these mutations and metastasis is more clearly understood.

In this work, LIMK1 was excluded from the final driver mutation list owing to its insignificant correlation with EMT markers. However, it is known that LIMK1 enhances tumor proliferation and metastasis *in vitro* and *in vivo* and is also involved in actin cytoskeleton dynamics for cancer invasiveness [30, 56, 57]. An interesting fact is that LIMK1 is considered as a non-Smad signaling regulator for EMT. LIMK1 transduces EMT-related signals without association with Smad [58]. Snail, Claudin-1 and ZEB1 are regulated by or closely cooperate with Smad for the control of EMT in response to transforming growth factor-β [59–62]. This explains why we observed little difference between the LIMK1 MT and WT expression on the level of Snail, Claudin-1 or ZEB1. Therefore, it is recommended that further research be undertaken to uncover the LIMK1-assciated signaling pathways in EMT.

The International Cancer Genome Consortium determined that 500 samples per cancer type would be required to detect somatic mutations that occur at a frequency of >3% [3]. Moreover, many more important drivers may be lurking in the places that we cannot understand precisely. These include copy number aberrations, large-genome rearrangements (also called structural variations) and non-coding regions [5]. Even if we succeed to discover low frequency driver mutations that are not previously recognized with relatively small number of samples, large-scale genomic study with sophisticated and comprehensive analysis is still required to fulfill mutational catalog for cancer metastasis. In literature, although other computational approaches have been proposed to integrate both genomics and transcriptomics data such as HyperModules [63], the pipeline we presented here is more reasonable and useful for identifying rare driver mutations. We noticed that the dynamics underlying tumor development have been taken into account by some computational approaches, that is, Dynamical Network Biomarkers [64], for identifying biomarkers. Here we do not consider the dynamic process as the samples in the two stages of breast cancer are not the same and the relatively small number of samples cannot provide dynamics signal for metastasis. In the future, with more data available, we will refine our approach to take into account the dynamics of tumors.

In this work, we assumed that the gene expression is mainly regulated by TFs despite other possible factors. The DEGs were supposed to be the consequence of metastasis and used as the signatures characterizing metastasis. The mutations that can influence the activities of TFs were considered as candidate driver mutations, where only the TFs regulating DEGs were considered. Here only the mutations detected in coding regions were considered, while many cancer mutations have been reported to be in the non-coding region [65]. With the whole-genome sequencing data available in the future, our approach can be extended to identify driver mutations detected in non-coding regions. In addition, we supposed that differential expression was caused owing to genetic mutations. However, the changes in gene expression may be caused by non-genetic perturbations, for example, external cellular stress, owing to the new metastatic microenvironments. In the future, with more comprehensive data emerging, our approach will be improved further to predict environmental factors influencing gene expression.

## Materials and Methods

### Sample collection

All of the cancer tissues and paired-normal tissues or blood of breast cancer patients were provided by the Seoul National University Hospital. Informed consent was obtained prior to sampling, and the study was approved by the Institutional Review Board of Seoul National University Hospital (IRB No 1109-007-376). All experiments were performed in accordance with relevant guidelines and regulations. The tissues were preserved in operation room within 30 min after removing from patients. Patients presenting with stage IV or who received neoadjuvant chemotherapy were excluded from the analysis. Breast tumor/normal tissue and blood samples were collected from 1998 to 2008. We followed up on them for years. We classified the patients into three different groups: ‘Local recurrent’, ‘Distant metastasis’, and ‘NED (no evidence of disease)’. Local recurrent patients were excluded from this research. Finally, samples of only 78 patients were used to avoid perturbations by other characteristics, such as age, molecular subtypes and adjuvant hormonal treatment. ‘Distant metastasis’ group included 22 patients who have experienced distant metastasis within 5 years after initial treatment. The samples from these 22 patients were used as ‘HRM’ samples. There were 56 patients who showed no sign of relapse and metastasis for at least 5 years in the ‘NED’ group, and their samples were used as ‘LRM’ samples.

### RNA and DNA extraction

Total RNA was isolated from breast cancer tissues using TRIzol reagent (Invitrogen, Grand Island, NY, USA). RNA yield was determined by a RiboGreen assay (Invitrogen) and NanoDrop ND1000 (ThermoFisher Scientific, Waltham, MA, USA) before quality assessment with the Agilent 2100 Bioanalyzer (Agilent Technologies, Santa Clara, CA, USA) according to the manufacturer’s instructions. Genomic DNA was extracted with the QIAamp DNA Mini Kit (Qiagen, Valencia, CA, USA). DNA integrity was verified by 0.8% agarose gel electrophoresis. Quality and quantity of DNA was measured using the NanoDrop Spectrophotometer and Quant-iT PicoGreen dsDNA Reagent and Kits (Invitrogen), respectively.

### Exome and RNA sequencing

Samples were prepared as an Illumina sequencing library (Illumina, San Diego, CA, USA), and the sequencing libraries were enriched for the desired target using the Illumina Exome Enrichment protocol. The captured libraries were sequenced using Illuminal HiSeq 2000 Sequencer. Raw sequencing data were aligned to UCSC hg19 (http://genome.ucsc.edu/) using BWA (http://genome.ucsc.edu/) and TopHat [66]. SAMTOOLS (http://samtools.sourceforge.net/) was used to detect the single-nucleotide polymorphisms and Indels [67]. To prevent miscalls that might be caused by duplicated sequencing errors, possible PCR duplicates were removed using Picard tools (http://picard.sourceforge.net/). The quality of the sequencing data was assessed by evaluating criteria such as on-target coverage, number of on-target genotypes and mean read depth of target regions.

### Detection of metastatic breast cancer-specific somatic alterations and DEGs

To identify somatic mutations, single-nucleotide variants and Indels that were also identified in the normal tissue/blood counterparts were removed. Known variants present at frequency >0.1 in the 1 000 Genome project (October 2011), dbSNP, were also excluded as they are assumed to be unrelated to breast cancer or metastasis. ‘Synonymous’ and ‘Unknown’ single-nucleotide variants were also excluded for further analysis. Only the mutations in the exons and splice site regions were considered. We found 53 candidate mutations according to the following criteria: (1) those found in >9% of HRM patients and (2) those not found in LRM patients. Here we applied the *t*-test (*P*-value<0.01) to the FPKM (fragments per kilobase of gene model per million mapped reads) values from RNA sequencing between HRM and LRM samples to detect DEGs. In addition, the mean expression value of each differential gene should follow twofold change.

### Mathematic model for detecting candidate driver mutations

To detect the most possible driver mutations underlying metastasis, we constructed a mathematic model that is able to identify the minimized set of mutations that cover as much samples as possible. Given the mutation matrix (m*n), each row represents a sample and each column a mutant gene, and the elements in the matrix are either 1 or 0. If the mutation occurs in one sample, the element is 1 and 0 otherwise. With the model below, we aimed to find *K* mutations that cover as much samples as possible but make sure the mutations are exclusive.

(1)Max∑j=1ncjxjM−λK⋅∑i=1n∑j=1,j≠incijxjxici⋅K,

$$s\mathrm{.t}.\;\sum_{j=1}^{n}x_{j}=K,x_{j}\in \{0,1\},$$

where *c*_*j*_ denotes the sum of entries in the column *j*, *c*_*ij*_ denotes the number of such rows 5 that is simultaneously covered by columns 5 and 5, that is, $\{k|a_{ki\_}\cdot a_{kj\_}=1\}$, 5 denotes the maximal value of *c*_*j*_ for all columns, 5 is a constant that specifies the number of columns to be selected and *λ* is a constant parameter to balance the coverage and exclusivity.

### Detection of pathways linking mutations to TFs regulating DEGs

The protein–protein interaction network was represented as a weighted undirected graph *G (V*, *E*, *W)*, where the vertices are proteins and *E* stands for the set of interactions between different proteins. In this work, *W* represents the interaction reliability between the corresponding proteins, which was defined by the Pearson’s correlation coefficient based on their transcriptional expression profiles under metastatic state:

(2)ωij=cov(X,Y)σxσy

where *X* and *Y* are the expression profiles of genes *i* and *j*, respectively, and cov(*X*,*Y*) is the covariance of the two variables, while *σ*_*x*_ and *σ*_*y*_ are their s.d.

Next our previously developed network flow model was utilized to detect the pathways bridging mutations and TFs. In brief, the model can be described as follows.

(3)Max{xi,yij,zij}∑i∈V∪{s,t}∑j∈V∪{s,t}wijyij−λ∑i∈V∑j∈Vyij

(4)s.t.yij⩽xi,

(5)yij⩽xj,

(6)∑j∈V∪{s,t}yij⩾1,ifiissort,

(7)∑j∈V∪{s,t}yij⩾2xi,ifiis\ notsort,

(8)∑j∈V∪{t}zij=R+1,

(9)∑i∈V∪{s,t}zij−∑k∈V∪{t}zjk=xj,forj∈V∪{t},

(10)∑i∈V∪{s,t}zij⩽(R+1)xj,forj∈V∪{t},

(11)xi=1,ifiissort,

(12)xi∈{0,1},i∈V∪{s,t},

(13)yij∈{0,1},i,j∈V∪{s,t},

(14)zij∈{0,1},i∈V∪{s,t},j∈V∪{t}

where a dummy node *s* denotes the source of signaling from mutant genes to the downstream pathways, *t* represents the set of TFs, *w*_*ij*_ is the weight of the edge *E(i,j)* in the undirected weighted network *G*, and *x*_*i*_ and *y*_*ij*_ are binary variables that, respectively, mean whether protein *i* and edge *E(i,j)* involved in the resultant pathway. The constraints *y*_*ij*_⩽*x*_*j*_ and *y*_*ij*_⩽*x*_*j*_ ensure that the interaction *E(i,j)* should be considered only when proteins *i* and *j* are both selected as components of the pathway. On the other hand, the constraint $\sum_{j}y_{ij}⩾1$ makes sure that each mutation or TF has at least one link to the other proteins, and $\sum_{i}y_{ij}⩾2x_{i}$ means that *x*_*i*_ has at least two linking edges once it is selected, thereby to ensure the connectivity of the resultant pathway. In addition, *Z*_*ij*_ denotes the number of units of flow from node *i* to node *j*, and *Z*_*ij*_=0 if there is no edge between *i* and *j* in the protein–protein interaction network. *R* is the upper bound of the size for the final pathway, where the constraint $\sum_{j\in V\cup \{t\}}z_{ij}=R+1$ means there are *R+1* units of flow entering the network from *s*. $\sum_{i\in V\cup \{s,t\}}z_{ij}-\sum_{k\in V\cup \{t\}}z_{jk}=x_{j}$ means one unit will leave the network if *j* is selected. The constraint $\sum_{i\in V\cup \{s,t\}}z_{ij}⩽(R+1)x_{j}$ ensures that once the protein *j* is selected as a component of the pathway, the sum of units entering *j* is no more than R+1. In short, Equations (11, 12, 13, 14) are used to make sure that there can be a path between nodes *s* and *t*. Finally, the parameter *λ* in the objective function controls the sparsity of the signaling pathway to be obtained. In addition, the weight for each pathway linking from mutant gene to one of its target TFs was defined as below.

(15)Pw=∑iwin

where *w*_*i*_ denotes the weight of the *i*th edge among the *n* edges in the pathway. For all the pathways starting from each mutant gene, the maximum weight of those pathways was used as the weight for the mutant gene. In this way, all the mutant genes can be ranked, and those top ranked mutant genes were more likely to affect the TFs regulating those DEGs and were therefore potential driver mutations.

### Antibodies

Anti-ZEB1 (3 396, 1:1000), Snail (3 879, 1:1 000) and Claudin-1 (13 255, 1:1 000) antibodies were obtained from Cell Signaling (Danvers, MA, USA). Anti-GFP (sc-9996, 1:1 000) antibody was obtained from Santa Cruz Biotechnology (Dallas, TX, USA). Anti-Flag (F3165, 1:10 000), β-actin (A1978, 1:10 000) antibodies were obtained from Sigma-Aldrich (St Louis, MO, USA). Goat anti-mouse IgG (H+L) (HRP) (31 430, 1:20 000), goat anti-rabbit IgG (H+L) (HRP) (31 460, 1:20 000) antibodies were obtained from Life Technologies (Waltham, MA, USA).

### Cell culture and transfection

MDA-MB-231 cells were cultured at RPMI 1690 with 10% heat-inactivated fetal bovine serum (HyClone, GE Healthcare Life Sciences, Logan, UT, USA), 100 U ml^−1^ penicillin and 100 μl ml^−1^ streptomycin (HyClone) in humidified incubator with 5% CO_2_. TurboFect transfection reagent (Life Technologies) was used for reverse transfection.

### Transwell migration assay

To determine the effect of each mutant on cell migration, transwell migration assay was performed by using 24-well Transwell chambers with polycarbonate membranes (8.0-μm pore size; Costar, Corning, NY, USA). Each WT- and mutant-transfected MDA-MB-231 cells were suspended in serum-free RPMI media and added to the upper compartment at 1×10^5^ cells per well. To the lower compartment, RPMI containing 5% fetal bovine serum was added. The cells were incubated for 6 h at 37 °C and in 5% CO_2_ incubator. Then they were fixed with 70% methanol for 15 min, washed with phosphate-buffered saline three times, stained with hematoxylin (Sigma Aldrich) for 10 min and washed with distilled water. After removing the non-migrant cells from the top face of the membrane with a cotton swab, the membranes were excised from the chamber and mounted with Gel Mount (Biomeda, Foster City, CA, USA). The migrant cells were counted with three randomly selected scopes in high-power fields (×10).

### Scratch wound-healing assay

To perform scratch wound-healing assay, CellPlayer Migration Assay System (ESSEN BioScience, Ann Arbor, MI, USA) was used. MDA-MB-231 cells were transfected with each of WT and mutant plasmid DNA and 2×10 ^4^ cells per well were seeded into collagen-coated 96-well ImageLock plate (ESSEN BioScience). After 95% cell confluency, a scratch was placed in middle of the wells using the WoundMaker (ESSEN BioScience). After washing twice with serum-free RPMI, the cells were incubated in RPMI containing 3% fetal bovine serum and the plate was put into IncuCyte FLR instrument (ESSEN BioScience). The cells were monitored for 48 h in IncuCyte FLR instrument and analyzed using the IncuCyte FLR 2011A software (ESSEN BioScience).

### Western blotting

Cells were lysed in lysis buffer (50 mM Tris-HCl at pH 7.4, 150 mM NaCl, 1 mM MgCl_2_, 1 mM EDTA, 0.5 mM EGTA, 0.5% Triton X-100, 0.1% sodium dodecyl sulfate, 0.5% Na-deoxycholate, protease inhibitor cocktail (Calbiochem, Merck Millipore Corporation, Darmstadt, Germany)) and incubated at 4 °C for 30 min. Equal amounts of protein were loaded, and sodium dodecyl sulfate–polyacrylamide gel electrophoresis was conducted. Proteins were transferred to polyvinylidene difluoride membrane, and immunoblotting was performed using a standard protocol.

### Data access

All sequencing files are available from the European Nucleotide Archive database (http://www.ebi.ac.uk/ena/data/view/PRJEB9083) and Biocon (ftp://ngs.biocon.re.kr/BreastCancer/).

## Figures and Tables

**Table 1:** Patient characteristics

*Information of HRM and LRM breast cancer patients*				
*Characteristic*	*HRM patients*		*LRM patients*	
	*No.*	*(%)*	*No.*	*(%)*
Overall	22	100.0	56	100.0
				
*Age, years*				
⩽39	6	27.3	15	26.8
40–49	2	9.1	12	21.4
50–59	7	31.8	20	35.7
⩾60	7	31.8	9	16.1
				
*Estrogen receptor status*				
Positive	10	45.5	24	42.9
Negative	12	54.5	32	57.1
				
*Progesterone receptor status*				
Positive	9	40.9	20	35.7
Negative	13	59.1	36	64.3
				
*HER2 status*				
Positive	3	13.6	7	12.5
Negative/unknown	19	86.4	49	87.5
				
*Adjuvant hormonal treatment*				
Yes	8	36.4	28	50.0
No/unknown	14	63.6	28	50.0
				
*Molecular subtype*				
Luminal	11	50.0	27	48.2
TNBC	7	31.8	18	32.1
HER2	1	4.5	5	8.9
Non-luminal	3	13.6	6	10.7

Abbreviations: HRM, high risk for distant metastasis; LRM, low risk for distant metastasis.
