# Supplementary material for: Integrative analysis of mutational and transcriptional profiles reveals driver mutations of metastatic breast cancers
Source: Cell Discov. 2016 Aug 30;2:16025–. doi: 10.1038/celldisc.2016.25 (PMC5004232; doi:10.1038/celldisc.2016.25)
Supplement: Supplementary Figure S1 [file celldisc201625-s1.pdf]

Supplementary Figure 1. Comparison of somatic mutations between HRM and LRM patients

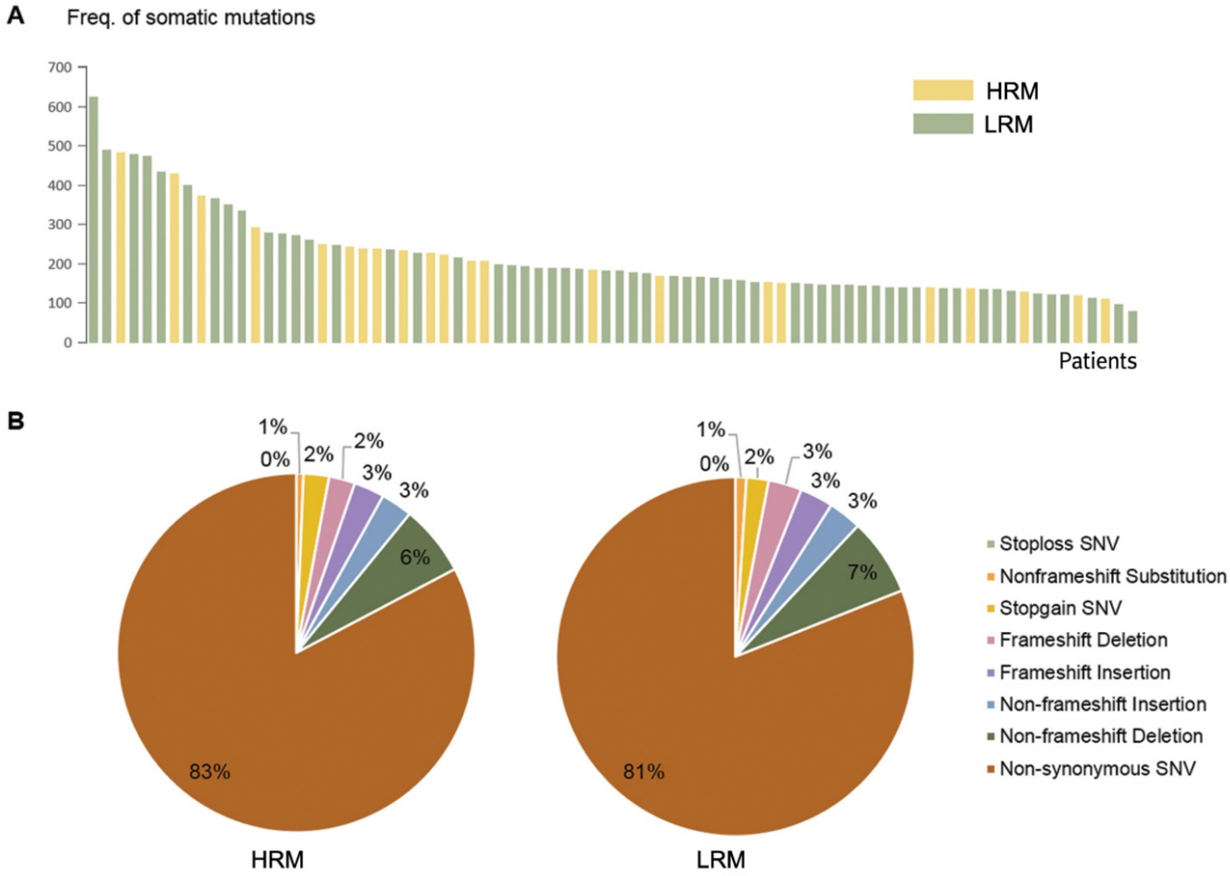

A. The frequencies of somatic mutations in 22 HRM (high-risk for distant metastasis) and 56 LRM (low-risk for distant metastasis) patients (Student t test P value =0.33).
B. Mutation types comparison between two groups.
