# Supplementary figures and images for: Integrative analysis of mutational and transcriptional profiles reveals driver mutations of metastatic breast cancers
Source: Cell Discov. 2016 Aug 30;2:16025–. doi: 10.1038/celldisc.2016.25 (PMC5004232; doi:10.1038/celldisc.2016.25)

**Supplementary Figure 2. Frequency comparison of cancer-related somatic mutations**

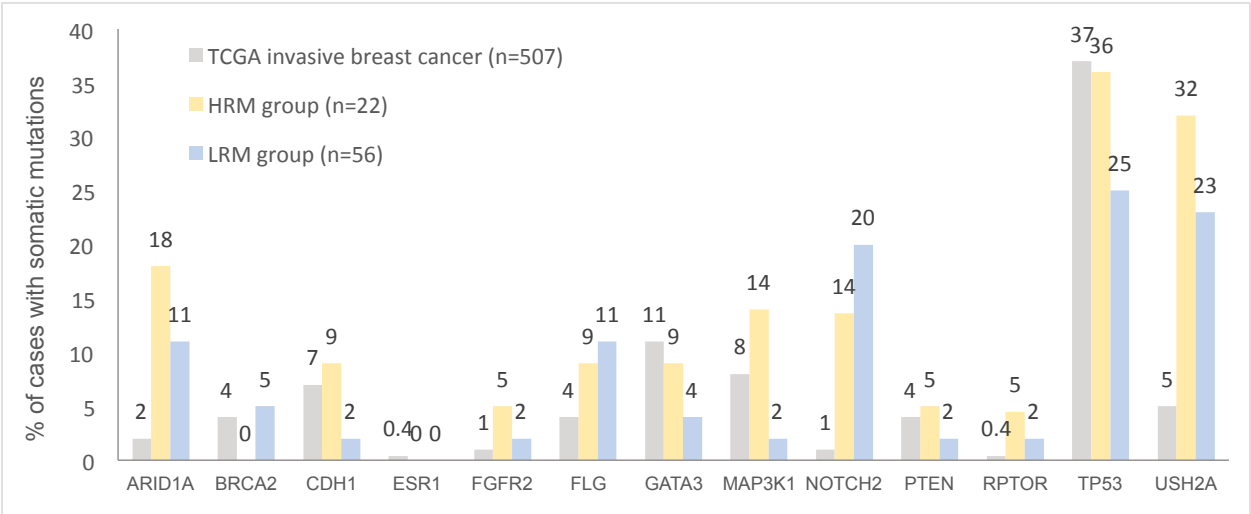

Supplement: Supplementary Figure S2 [file celldisc201625-s2.pdf]

Supplementary Figure 4. Wound healing assay results of nine mutations

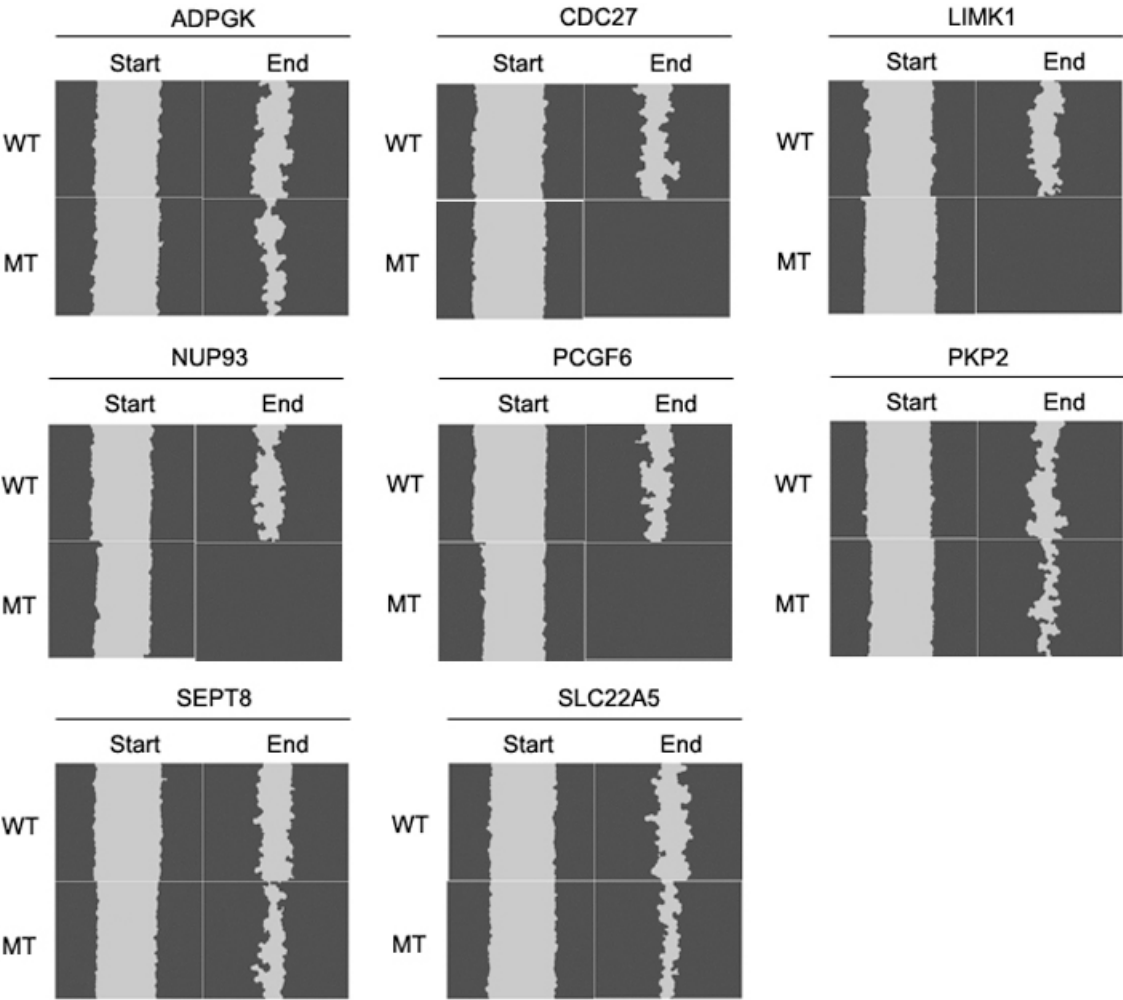

Supplement: Supplementary Figure S4 [file celldisc201625-s4.pdf]
