# Supplementary material for: Integrative analysis of mutational and transcriptional profiles reveals driver mutations of metastatic breast cancers
Source: Cell Discov. 2016 Aug 30;2:16025–. doi: 10.1038/celldisc.2016.25 (PMC5004232; doi:10.1038/celldisc.2016.25)

Supplementary Figure 3. The distribution of HRM-specific mutations across HRM samples

The red block indicates that a mutation has been detected in the corresponding sample.

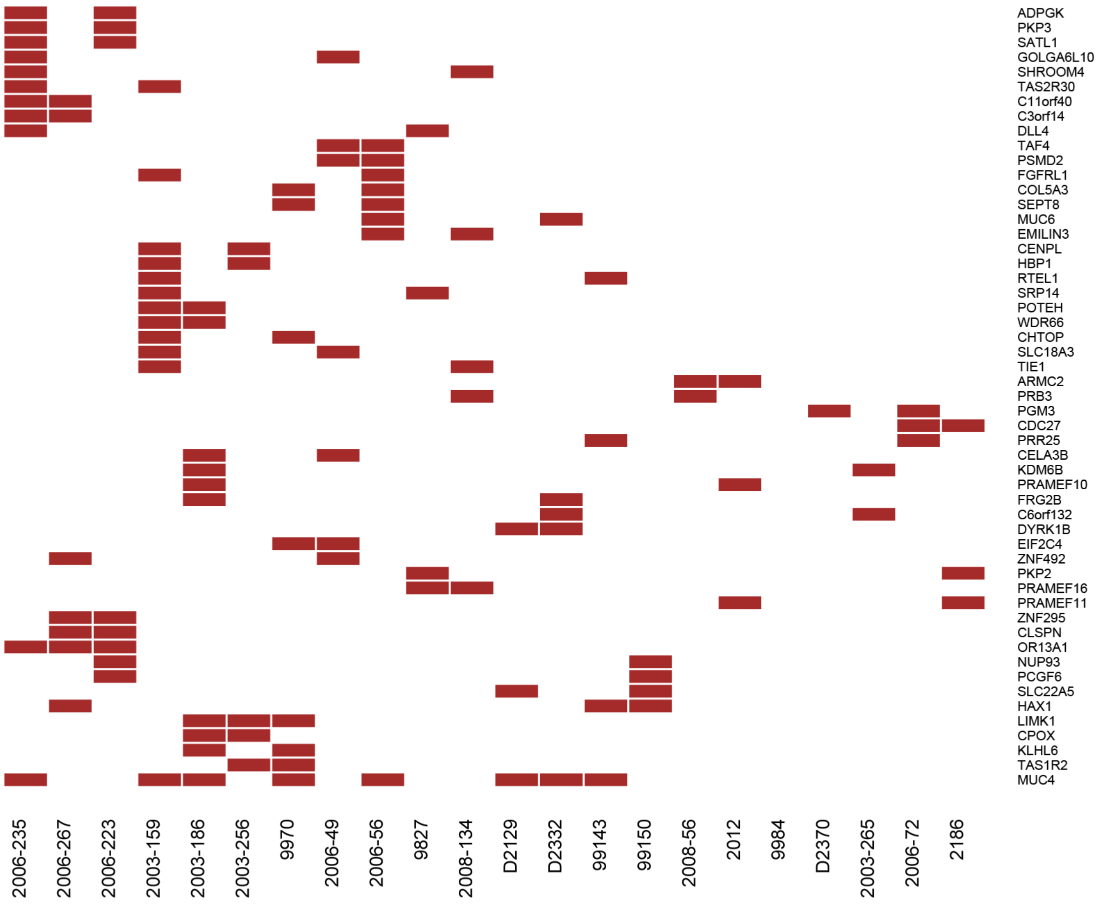

Supplement: Supplementary Figure S3 [file celldisc201625-s3.pdf]
