# Supplementary material for: Integrative analysis of mutational and transcriptional profiles reveals driver mutations of metastatic breast cancers
Source: Cell Discov. 2016 Aug 30;2:16025–. doi: 10.1038/celldisc.2016.25 (PMC5004232; doi:10.1038/celldisc.2016.25)

Supplementary Figure 5. Predicted signaling pathways altered by five driver mutations

The shortest pathways linking mutations to all associated TFs.

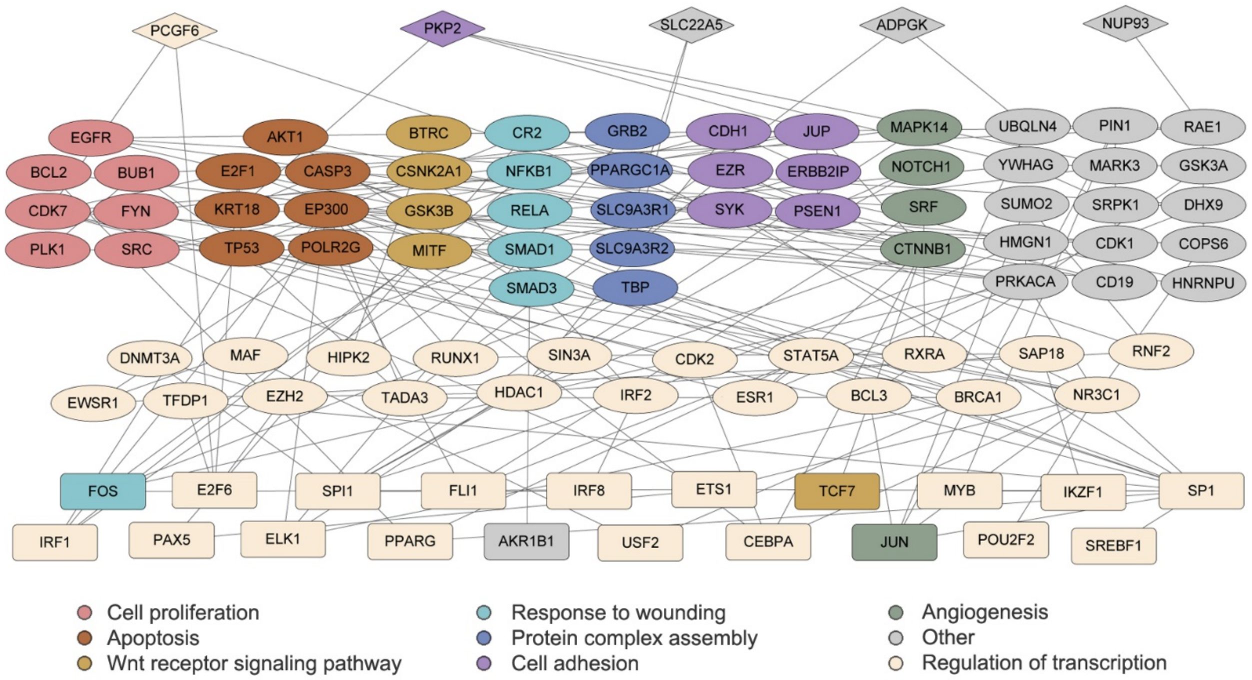

Supplement: Supplementary Figure S5 [file celldisc201625-s5.pdf]
