# Supplementary material for: Integrative analysis of mutational and transcriptional profiles reveals driver mutations of metastatic breast cancers
Source: Cell Discov. 2016 Aug 30;2:16025–. doi: 10.1038/celldisc.2016.25 (PMC5004232; doi:10.1038/celldisc.2016.25)
Supplement: Supplementary Table S1 [file celldisc201625-s6.pdf]

## Supplementary Table 1. Patient information

* POS: Positive, NEG: Negative, UN: Unknown

| Class | Sample Number | ER | PR | HER2 | Molecular Subtype | Age | T Stage | Histological Grade | p53 | Bcl-2 | Ki-67 | Hormonal Treatment |
|---|---|---|---|---|---|---|---|---|---|---|---|---|
| HRM | 2008-134 | 0 | 0 | 0 | TNBC | 67 | UN | UN | UN | UN | UN | UN |
| HRM | 2006-72 | 0 | 0 | 0 | TNBC | 39 | UN | UN | UN | UN | UN | UN |
| HRM | 2006-56 | 0 | 0 | 0 | TNBC | 62 | UN | UN | UN | UN | UN | UN |
| HRM | 2003-256 | 0 | 0 | 0 | TNBC | 55 | 2 | IIB | POS | NEG | POS | 0 |
| HRM | 2003-265 | 0 | 0 | 0 | Non-luminal | 40 | UN | UN | UN | UN | UN | UN |
| HRM | 2003-159 | 0 | 0 | 0 | TNBC | 43 | 2 | IIB | NEG | POS | POS | 0 |
| HRM | 2003-186 | 0 | 0 | 0 | TNBC | 51 | 2 | IIIC | NEG | NEG | POS | 0 |
| HRM | 2006-223 | 1 | 1 | 1 | Luminal | 62 | 2 | IIA | NEG | POS | POS | 1 |
| HRM | 2006-235 | 1 | 1 | 0 | Luminal | 36 | 2 | IIA | POS | POS | POS | 1 |
| HRM | 2006-267 | 1 | 0 | 1 | Luminal | 60 | 2 | IIA | POS | POS | POS | 1 |
| HRM | 2006-49 | 1 | 1 | 0 | Luminal | 37 | 2 | IIA | NEG | POS | POS | 1 |
| HRM | 2008-56 | 0 | 0 | 1 | HER2 | 72 | UN | UN | UN | UN | UN | UN |
| HRM | 2012 | 1 | 1 | UN | Luminal | 37 | 1c | IIA | POS | POS | POS | 1 |
| HRM | D2129 | 0 | 0 | 0 | TNBC | 32 | 3 | IIIA | POS | NEG | UN | 0 |
| HRM | 2186 | 1 | 1 | 0 | Luminal | 63 | 2 | IIB | POS | NEG | POS | 1 |
| HRM | D2332 | 0 | 1 | 0 | Luminal | 56 | 1c | I | POS | POS | POS | 1 |
| HRM | D2370 | 1 | 1 | 0 | Luminal | 37 | 2 | IIB | NEG | NEG | POS | 1 |
| HRM | 9827 | 1 | 0 | 0 | Luminal | 51 | UN | UN | UN | UN | UN | UN |
| HRM | 99143 | 1 | 1 | 0 | Luminal | 63 | UN | UN | UN | UN | UN | UN |
| HRM | 99150 | 1 | 1 | 0 | Luminal | 51 | UN | UN | UN | UN | UN | UN |
| HRM | 9970 | 0 | 0 | 0 | Non-luminal | 58 | UN | UN | UN | UN | UN | UN |
| HRM | 9984 | 0 | 0 | 0 | Non-luminal | 55 | UN | UN | UN | UN | UN | UN |
| LRM | D2006-84 | 0 | 0 | 0 | TNBC | 40 | 3 | IIB | POS | POS | POS | 0 |
| LRM | 2003-303 | 0 | 0 | UN | Non-luminal | 43 | 1c | IIA | POS | POS | POS | 0 |
| LRM | 2107 | 1 | 1 | 0 | Luminal | 54 | 2 | IIA | POS | NEG | UN | 1 |
| LRM | D2096 | 0 | 0 | 0 | TNBC | 58 | 1c | UN | NEG | NEG | POS | 0 |
| LRM | 2083 | 1 | 1 | 1 | Luminal | 65 | 1c | I | POS | POS | POS | 1 |
| LRM | 2062 | 1 | 1 | 1 | Luminal | 51 | 2 | IIB | POS | POS | POS | 1 |
| LRM | 2051 | 0 | 0 | 0 | TNBC | 45 | 1c | I | NEG | NEG | POS | 0 |
| LRM | 2060 | 0 | 1 | 0 | Luminal | 51 | 2 | IIB | UN | UN | UN | 1 |
| LRM | 2150 | 0 | 0 | UN | Non-luminal | 45 | 2 | IIA | NEG | POS | POS | 0 |
| LRM | 2146 | 0 | 0 | 1 | HER2 | 69 | 2 | IIA | UN | UN | UN | 0 |
| LRM | 2142 | 0 | 0 | 0 | TNBC | 43 | 2 | IIA | POS | NEG | POS | 0 |
| LRM | 2172 | 0 | 0 | 0 | TNBC | 37 | 2 | IIB | POS | NEG | POS | 1 |
| LRM | D2224 | 1 | 1 | 0 | Luminal | 51 | 1b | I | POS | NEG | POS | 1 |
| LRM | 2222 | 1 | UN | UN | Luminal | 51 | 2 | IIA | POS | POS | POS | 1 |
| LRM | 2214 | 1 | 1 | 0 | Luminal | 54 | 1c | I | POS | NEG | POS | 1 |
| LRM | 2234 | 1 | 0 | 0 | Luminal | 39 | 2 | IIA | NEG | NEG | POS | 1 |
| LRM | 2189 | 0 | 0 | 0 | TNBC | 52 | 1c | I | NEG | POS | POS | 0 |
| LRM | 2188 | 1 | 0 | 0 | Luminal | 38 | 1b | I | POS | POS | POS | 1 |
| LRM | 2195 | 1 | 0 | 0 | Luminal | 53 | 1c | IIA | NEG | NEG | POS | 1 |
| LRM | D2268 | 0 | 0 | 0 | TNBC | 34 | 1c | I | POS | POS | POS | 0 |
| LRM | 2243 | 1 | 1 | UN | Luminal | 41 | 2 | IIA | POS | NEG | POS | 1 |
| LRM | D2303 | 0 | 0 | 0 | TNBC | 52 | 2 | IIA | NEG | POS | POS | 0 |
| LRM | 2242-2 | 1 | 1 | 0 | Luminal | 56 | 1c | UN | NEG | NEG | POS | 1 |
| LRM | 2183 | 1 | 1 | 0 | Luminal | 39 | 2 | IIB | POS | NEG | POS | 1 |
| LRM | 2202 | 0 | 0 | 1 | HER2 | 37 | 2 | IIA | NEG | NEG | POS | 0 |
| LRM | 2198 | 0 | 0 | 1 | HER2 | 36 | 2 | IIB | POS | NEG | POS | 0 |
| LRM | 2194 | 1 | 0 | 0 | Luminal | 56 | 2 | IIB | POS | POS | POS | 1 |

| | | | | | | | | | | | | |
|---|---|---|---|---|---|---|---|---|---|---|---|---|
| LRM | 2304 | 1 | 1 | 0 | Luminal | 39 | 2 | IIB | NEG | POS | POS | 1 |
| LRM | D2247 | 0 | 0 | UN | Non-luminal | 53 | 2 | IIB | POS | POS | POS | 0 |
| LRM | 2281 | 1 | 1 | UN | Luminal | 40 | 2 | IIA | NEG | NEG | POS | 1 |
| LRM | 2317 | 1 | 1 | 0 | Luminal | 39 | 2 | IIB | POS | POS | POS | 1 |
| LRM | 2358 | 1 | 1 | 0 | Luminal | 63 | 2 | IIA | NEG | NEG | POS | 1 |
| LRM | 2357 | 0 | 0 | UN | Non-luminal | 39 | 2 | IIIA | POS | POS | POS | 0 |
| LRM | 2359 | 0 | 0 | 0 | TNBC | 45 | 2 | IIA | POS | NEG | POS | 0 |
| LRM | 2379 | 0 | 0 | 1 | HER2 | 36 | 2 | IIA | NEG | NEG | POS | 0 |
| LRM | 2378 | 0 | 0 | 0 | TNBC | 41 | 1c | IIA | POS | NEG | POS | 0 |
| LRM | 2006-315 | 0 | 0 | 0 | TNBC | 69 | 1c | IIA | POS | POS | POS | 0 |
| LRM | 2006-313 | 0 | 0 | 1 | HER2 | 63 | 2 | IIA | POS | NEG | POS | 0 |
| LRM | 2006-187 | 0 | 0 | 0 | TNBC | 53 | 1c | IIA | POS | POS | POS | 0 |
| LRM | 2003-237 | 1 | 1 | 0 | Luminal | 61 | 2 | IIIA | POS | POS | POS | 1 |
| LRM | 2006-174 | 0 | 0 | 0 | TNBC | 42 | 2 | IIIA | POS | POS | POS | 1 |
| LRM | 2006-225 | 0 | 1 | 0 | Luminal | 51 | 2 | IIA | POS | NEG | POS | 1 |
| LRM | 2006-241 | 0 | 1 | 0 | Luminal | 52 | 2 | IIB | POS | NEG | POS | 1 |
| LRM | 2006-149 | 1 | 1 | 0 | Luminal | 73 | 2 | IIB | POS | POS | POS | 1 |
| LRM | 2006-316 | 0 | 0 | 0 | TNBC | 30 | 1c | I | POS | POS | POS | 0 |
| LRM | 2006-16 | 1 | 1 | 0 | Luminal | 38 | 2 | IIIA | POS | POS | POS | 1 |
| LRM | 2006-173 | 0 | 0 | UN | Non-luminal | 58 | 2 | IIA | POS | POS | POS | 0 |
| LRM | 2006-38 | 1 | 1 | UN | Luminal | 64 | 2 | IIB | POS | NEG | POS | 1 |
| LRM | 2006-21 | 0 | 0 | 0 | TNBC | 71 | 2 | IIIA | POS | NEG | POS | 0 |
| LRM | 2006-82 | 1 | 1 | 0 | Luminal | 43 | 2 | IIIA | NEG | POS | POS | 1 |
| LRM | D2008-10 | 0 | 0 | 0 | TNBC | 55 | 2 | UN | NEG | NEG | POS | 0 |
| LRM | D2008-118 | 1 | 0 | 0 | Luminal | 39 | 3 | IIIA | POS | POS | POS | 1 |
| LRM | D2008-112 | 0 | 0 | 0 | TNBC | 35 | 2 | IIB | POS | NEG | POS | 0 |
| LRM | 9829 | 0 | 0 | 0 | TNBC | 59 | UN | UN | UN | UN | UN | UN |
| LRM | 9964 | 0 | 0 | 0 | Non-luminal | 46 | UN | UN | UN | UN | UN | UN |
| LRM | 9653 | 1 | 0 | UN | Luminal | 53 | UN | UN | UN | UN | UN | UN |
