# Supplementary material for: Integrative analysis of mutational and transcriptional profiles reveals driver mutations of metastatic breast cancers
Source: Cell Discov. 2016 Aug 30;2:16025–. doi: 10.1038/celldisc.2016.25 (PMC5004232; doi:10.1038/celldisc.2016.25)
Supplement: Supplementary Table S2 [file celldisc201625-s7.pdf]

Supplementary Table 2. Exome Stats

| Group | Data_Sample | CapKit | Total_reads | Total_yield | Average_read_length |
|---|---|---|---|---|---|
| Meta | 2003-159 | TruSeq | 71806624 | 7252469024 | 101 |
| Meta | 2003-186 | TruSeq | 71431568 | 7214588368 | 101 |
| Meta | 2003-256 | TruSeq | 67746608 | 6842407408 | 101 |
| Meta | 2003-265 | TruSeq | 107289452 | 10836234652 | 101 |
| Meta | 2006-223 | TruSeq | 124465416 | 12571007016 | 101 |
| Meta | 2006-235 | TruSeq | 42917684 | 4334686084 | 101 |
| Meta | 2006-267 | TruSeq | 72791540 | 7351945540 | 101 |
| Meta | 2006-49 | TruSeq | 63355022 | 6398857222 | 101 |
| Meta | 2006-56 | TruSeq | 120148366 | 12134984966 | 101 |
| Meta | 2006-72 | TruSeq | 95110724 | 9606183124 | 101 |
| Meta | 2008-134 | TruSeq | 121307940 | 12252101940 | 101 |
| Meta | 2008-56 | TruSeq | 139524402 | 14091964602 | 101 |
| Meta | 2012 | TruSeq | 67694974 | 6837192374 | 101 |
| Meta | 2186 | TruSeq | 75429684 | 7618398084 | 101 |
| Meta | 9827 | TruSeq | 96312624 | 9727575024 | 101 |
| Meta | 99143 | TruSeq | 95297328 | 9625030128 | 101 |
| Meta | 99150 | TruSeq | 89868026 | 9076670626 | 101 |
| Meta | 9970 | TruSeq | 104601038 | 10564704838 | 101 |
| Meta | 9984 | TruSeq | 84835320 | 8568367320 | 101 |
| Meta | D2129 | TruSeq | 71411018 | 7212512818 | 101 |
| Meta | D2332 | TruSeq | 149217302 | 15070947502 | 101 |
| Meta | D2370 | TruSeq | 68005646 | 6868570246 | 101 |
| Non-Meta | 2003-237 | TruSeq | 66747184 | 6741465584 | 101 |
| Non-Meta | 2003-303 | TruSeq | 274294734 | 27703768134 | 101 |
| Non-Meta | 2006-149 | TruSeq | 71203584 | 7191561984 | 101 |
| Non-Meta | 2006-16 | TruSeq | 63959354 | 6459894754 | 101 |
| Non-Meta | 2006-173 | TruSeq | 65060364 | 6571096764 | 101 |
| Non-Meta | 2006-174 | TruSeq | 75272122 | 7602484322 | 101 |
| Non-Meta | 2006-187 | TruSeq | 66586432 | 6725229632 | 101 |
| Non-Meta | 2006-21 | TruSeq | 66671754 | 6733847154 | 101 |
| Non-Meta | 2006-225 | TruSeq | 142889558 | 14431845358 | 101 |
| Non-Meta | 2006-241 | TruSeq | 66686306 | 6735316906 | 101 |
| Non-Meta | 2006-313 | TruSeq | 118802790 | 11999081790 | 101 |
| Non-Meta | 2006-315 | TruSeq | 92977146 | 9390691746 | 101 |
| Non-Meta | 2006-316 | TruSeq | 86935254 | 8780460654 | 101 |
| Non-Meta | 2006-38 | TruSeq | 62203378 | 6282541178 | 101 |
| Non-Meta | 2006-82 | TruSeq | 65404370 | 6605841370 | 101 |
| Non-Meta | 2051 | TruSeq | 63903874 | 6454291274 | 101 |
| Non-Meta | 2060 | TruSeq | 74434504 | 7517884904 | 101 |
| Non-Meta | 2062 | TruSeq | 60625852 | 6123211052 | 101 |
| Non-Meta | 2083 | TruSeq | 63221230 | 6385344230 | 101 |
| Non-Meta | 2107 | TruSeq | 65908270 | 6656735270 | 101 |
| Non-Meta | 2142 | TruSeq | 68623874 | 6931011274 | 101 |
| Non-Meta | 2146 | TruSeq | 70653476 | 7136001076 | 101 |

| | | | | | |
|---|---|---|---|---|---|
| Non-Meta | 2150 | TruSeq | 65283598 | 6593643398 | 101 |
| Non-Meta | 2172 | TruSeq | 115135026 | 11628637626 | 101 |
| Non-Meta | 2183 | TruSeq | 95016516 | 9596668116 | 101 |
| Non-Meta | 2188 | TruSeq | 68920978 | 6961018778 | 101 |
| Non-Meta | 2189 | TruSeq | 72205654 | 7292771054 | 101 |
| Non-Meta | 2194 | TruSeq | 68658006 | 6934458606 | 101 |
| Non-Meta | 2195 | TruSeq | 97252636 | 9822516236 | 101 |
| Non-Meta | 2198 | TruSeq | 71723840 | 7244107840 | 101 |
| Non-Meta | 2202 | TruSeq | 69433814 | 7012815214 | 101 |
| Non-Meta | 2214 | TruSeq | 248051486 | 25053200086 | 101 |
| Non-Meta | 2222 | TruSeq | 61514562 | 6212970762 | 101 |
| Non-Meta | 2234 | TruSeq | 63535838 | 6417119638 | 101 |
| Non-Meta | 2242-2 | TruSeq | 76047250 | 7680772250 | 101 |
| Non-Meta | 2243 | TruSeq | 67180240 | 6785204240 | 101 |
| Non-Meta | 2281 | TruSeq | 61334316 | 6194765916 | 101 |
| Non-Meta | 2304 | TruSeq | 67641824 | 6831824224 | 101 |
| Non-Meta | 2317 | TruSeq | 72207034 | 7292910434 | 101 |
| Non-Meta | 2357 | TruSeq | 69014828 | 6970497628 | 101 |
| Non-Meta | 2358 | TruSeq | 60245342 | 6084779542 | 101 |
| Non-Meta | 2359 | TruSeq | 67930688 | 6860999488 | 101 |
| Non-Meta | 2378 | TruSeq | 66850376 | 6751887976 | 101 |
| Non-Meta | 2379 | TruSeq | 78776448 | 7956421248 | 101 |
| Non-Meta | 9653 | TruSeq | 100237252 | 10123962452 | 101 |
| Non-Meta | 9829 | TruSeq | 102951148 | 10398065948 | 101 |
| Non-Meta | 9964 | TruSeq | 107980818 | 10906062618 | 101 |
| Non-Meta | D2006-84 | TruSeq | 109043482 | 11013391682 | 101 |
| Non-Meta | D2008-10 | TruSeq | 65332634 | 6598596034 | 101 |
| Non-Meta | D2008-112 | TruSeq | 62429146 | 6305343746 | 101 |
| Non-Meta | D2008-118 | TruSeq | 69083774 | 6977461174 | 101 |
| Non-Meta | D2096 | TruSeq | 102940120 | 10396952120 | 101 |
| Non-Meta | D2224 | TruSeq | 103143650 | 10417508650 | 101 |
| Non-Meta | D2247 | TruSeq | 69672480 | 7036920480 | 101 |
| Non-Meta | D2268 | TruSeq | 66455362 | 6711991562 | 101 |
| Non-Meta | D2303 | TruSeq | 82447696 | 8327217296 | 101 |

| Target_regions | Average_throughput_depth_of_target_regions | Initial_mappable_reads_mapped_to_human_genome |
|---|---|---|
| 62085286 | 117 | 71806624 |
| 62085286 | 116 | 71431568 |
| 62085286 | 110 | 67746608 |
| 62085286 | 175 | 107147652 |
| 62085286 | 203 | 124465416 |
| 62085286 | 70 | 42917684 |
| 62085286 | 118 | 72791540 |
| 62085286 | 103 | 63355022 |
| 62085286 | 196 | 119946126 |
| 62085286 | 155 | 94936864 |
| 62085286 | 197 | 121133716 |
| 62085286 | 227 | 139310920 |
| 62085286 | 110 | 67694974 |
| 62085286 | 123 | 75429684 |
| 62085286 | 157 | 96152000 |
| 62085286 | 155 | 95176234 |
| 62085286 | 146 | 89727332 |
| 62085286 | 170 | 104365584 |
| 62085286 | 138 | 84728156 |
| 62085286 | 116 | 71411018 |
| 62085286 | 243 | 149217302 |
| 62085286 | 111 | 68005646 |
| 62085286 | 109 | 66747184 |
| 62085286 | 446 | 273613768 |
| 62085286 | 116 | 71203584 |
| 62085286 | 104 | 63959354 |
| 62085286 | 106 | 65060364 |
| 62085286 | 123 | 75272122 |
| 62085286 | 108 | 66586432 |
| 62085286 | 109 | 66671754 |
| 62085286 | 233 | 142889558 |
| 62085286 | 109 | 66686306 |
| 62085286 | 193 | 118802790 |
| 62085286 | 151 | 92977146 |
| 62085286 | 141 | 86935254 |
| 62085286 | 101 | 62203378 |
| 62085286 | 106 | 65404370 |
| 62085286 | 104 | 63903874 |
| 62085286 | 121 | 74434504 |
| 62085286 | 99 | 60625852 |
| 62085286 | 103 | 63221230 |
| 62085286 | 107 | 65908270 |
| 62085286 | 112 | 68623874 |
| 62085286 | 115 | 70653476 |

| | | |
|---|---|---|
| 62085286 | 106 | 65283598 |
| 62085286 | 187 | 115135026 |
| 62085286 | 155 | 95016516 |
| 62085286 | 112 | 68920978 |
| 62085286 | 118 | 72205654 |
| 62085286 | 112 | 68658006 |
| 62085286 | 158 | 97252636 |
| 62085286 | 117 | 71723840 |
| 62085286 | 113 | 69433814 |
| 62085286 | 404 | 242636962 |
| 62085286 | 100 | 61514562 |
| 62085286 | 103 | 63535838 |
| 62085286 | 124 | 76047250 |
| 62085286 | 109 | 67180240 |
| 62085286 | 100 | 61334316 |
| 62085286 | 110 | 67641824 |
| 62085286 | 118 | 72207034 |
| 62085286 | 112 | 69014828 |
| 62085286 | 98 | 60245342 |
| 62085286 | 111 | 67930688 |
| 62085286 | 109 | 66850376 |
| 62085286 | 128 | 78776448 |
| 62085286 | 163 | 100022238 |
| 62085286 | 168 | 102781852 |
| 62085286 | 176 | 107810456 |
| 62085286 | 177 | 109043482 |
| 62085286 | 106 | 65332634 |
| 62085286 | 102 | 62429146 |
| 62085286 | 112 | 69083774 |
| 62085286 | 168 | 102940120 |
| 62085286 | 168 | 102981652 |
| 62085286 | 113 | 69672480 |
| 62085286 | 108 | 66455362 |
| 62085286 | 134 | 82447696 |

| Initial_mappable_reads_out_of_total_reads | Non_redundant_reads_de_duplicated_by_Picard_tools | Non_redundant_reads_out_of_initial_mappable_reads |
|---|---|---|
| 100.00% | 58967621 | 82.10% |
| 100.00% | 57896280 | 81.10% |
| 100.00% | 55321621 | 81.70% |
| 99.90% | 71362504 | 66.60% |
| 100.00% | 104784962 | 84.20% |
| 100.00% | 41104537 | 95.80% |
| 100.00% | 61777104 | 84.90% |
| 100.00% | 53116170 | 83.80% |
| 99.80% | 86354030 | 72.00% |
| 99.80% | 59198076 | 62.40% |
| 99.90% | 86234730 | 71.20% |
| 99.80% | 82611050 | 59.30% |
| 100.00% | 53571890 | 79.10% |
| 100.00% | 57253340 | 75.90% |
| 99.80% | 51841255 | 53.90% |
| 99.90% | 55806455 | 58.60% |
| 99.80% | 54705247 | 61.00% |
| 99.80% | 45503157 | 43.60% |
| 99.90% | 55293993 | 65.30% |
| 100.00% | 59659664 | 83.50% |
| 100.00% | 86263490 | 57.80% |
| 100.00% | 56422986 | 83.00% |
| 100.00% | 55542068 | 83.20% |
| 99.80% | 111774834 | 40.90% |
| 100.00% | 62725076 | 88.10% |
| 100.00% | 52439080 | 82.00% |
| 100.00% | 56220447 | 86.40% |
| 100.00% | 66453390 | 88.30% |
| 100.00% | 54425490 | 81.70% |
| 100.00% | 56034326 | 84.00% |
| 100.00% | 115626512 | 80.90% |
| 100.00% | 55196703 | 82.80% |
| 100.00% | 90197302 | 75.90% |
| 100.00% | 74595841 | 80.20% |
| 100.00% | 57010800 | 65.60% |
| 100.00% | 51666791 | 83.10% |
| 100.00% | 55355274 | 84.60% |
| 100.00% | 48829548 | 76.40% |
| 100.00% | 59573216 | 80.00% |
| 100.00% | 48082371 | 79.30% |
| 100.00% | 51919557 | 82.10% |
| 100.00% | 54342918 | 82.50% |
| 100.00% | 55393806 | 80.70% |
| 100.00% | 56937424 | 80.60% |

| | | |
|---|---|---|
| 100.00% | 54261428 | 83.10% |
| 100.00% | 90394531 | 78.50% |
| 100.00% | 71135567 | 74.90% |
| 100.00% | 52268137 | 75.80% |
| 100.00% | 54993338 | 76.20% |
| 100.00% | 52817742 | 76.90% |
| 100.00% | 64885970 | 66.70% |
| 100.00% | 58268213 | 81.20% |
| 100.00% | 56630049 | 81.60% |
| 97.80% | 148111821 | 61.00% |
| 100.00% | 52455449 | 85.30% |
| 100.00% | 51005760 | 80.30% |
| 100.00% | 47549637 | 62.50% |
| 100.00% | 51979994 | 77.40% |
| 100.00% | 47377953 | 77.20% |
| 100.00% | 54955914 | 81.20% |
| 100.00% | 56655839 | 78.50% |
| 100.00% | 56805892 | 82.30% |
| 100.00% | 47213767 | 78.40% |
| 100.00% | 56767537 | 83.60% |
| 100.00% | 56372463 | 84.30% |
| 100.00% | 67291732 | 85.40% |
| 99.80% | 60698755 | 60.70% |
| 99.80% | 66145224 | 64.40% |
| 99.80% | 73292368 | 68.00% |
| 100.00% | 76913901 | 70.50% |
| 100.00% | 54410781 | 83.30% |
| 100.00% | 46117390 | 73.90% |
| 100.00% | 50948113 | 73.70% |
| 100.00% | 86752827 | 84.30% |
| 99.80% | 69025150 | 67.00% |
| 100.00% | 50849130 | 73.00% |
| 100.00% | 47563993 | 71.60% |
| 100.00% | 56541269 | 68.60% |

| Non_redundant_unique_reads_uniquely_mapped_to_human_genome | Non_redundant_unique_reads_out_of_non_redundant_reads |
|---|---|
| 52494793 | 89.00% |
| 50985791 | 88.10% |
| 49293503 | 89.10% |
| 64413236 | 90.30% |
| 90107868 | 86.00% |
| 36893695 | 89.80% |
| 55640956 | 90.10% |
| 47346770 | 89.10% |
| 77614449 | 89.90% |
| 53320792 | 90.10% |
| 77351250 | 89.70% |
| 74037214 | 89.60% |
| 47517133 | 88.70% |
| 50552982 | 88.30% |
| 46892833 | 90.50% |
| 50471114 | 90.40% |
| 49337731 | 90.20% |
| 40393929 | 88.80% |
| 49986337 | 90.40% |
| 53549048 | 89.80% |
| 76956519 | 89.20% |
| 50313053 | 89.20% |
| 49540391 | 89.20% |
| 100944670 | 90.30% |
| 56483829 | 90.00% |
| 46974148 | 89.60% |
| 50309918 | 89.50% |
| 59830843 | 90.00% |
| 48534923 | 89.20% |
| 50047678 | 89.30% |
| 94594270 | 81.80% |
| 49616058 | 89.90% |
| 80609175 | 89.40% |
| 66265188 | 88.80% |
| 50911752 | 89.30% |
| 46094961 | 89.20% |
| 49497253 | 89.40% |
| 43301027 | 88.70% |
| 53510030 | 89.80% |
| 42919961 | 89.30% |
| 46482467 | 89.50% |
| 48654249 | 89.50% |
| 49405923 | 89.20% |
| 50331522 | 88.40% |

| | |
|---|---|
| 48436497 | 89.30% |
| 80481847 | 89.00% |
| 63459530 | 89.20% |
| 46163311 | 88.30% |
| 48547479 | 88.30% |
| 46855564 | 88.70% |
| 57437212 | 88.50% |
| 51974105 | 89.20% |
| 50303206 | 88.80% |
| 131892657 | 89.00% |
| 46740014 | 89.10% |
| 45354869 | 88.90% |
| 42977803 | 90.40% |
| 46153662 | 88.80% |
| 42089573 | 88.80% |
| 48746166 | 88.70% |
| 50456906 | 89.10% |
| 50963607 | 89.70% |
| 42045470 | 89.10% |
| 50819835 | 89.50% |
| 50562732 | 89.70% |
| 60351115 | 89.70% |
| 53953116 | 88.90% |
| 59231881 | 89.50% |
| 64919145 | 88.60% |
| 68984433 | 89.70% |
| 48683466 | 89.50% |
| 41709590 | 90.40% |
| 45375896 | 89.10% |
| 78109467 | 90.00% |
| 62011784 | 89.80% |
| 45247240 | 89.00% |
| 42154562 | 88.60% |
| 50054713 | 88.50% |

| On_target_reads_reads_mapped_to_target_regions | On_target_reads_out_of_non_redundant_unique_reads | Coverage_of_target_regions_more_than_1X |
|---|---|---|
| 37199895 | 70.90% | 93.70% |
| 35511032 | 69.60% | 94.10% |
| 33568115 | 68.10% | 94.70% |
| 45194707 | 70.20% | 94.20% |
| 59425675 | 65.90% | 97.60% |
| 25731228 | 69.70% | 94.10% |
| 40388706 | 72.60% | 94.50% |
| 32600009 | 68.90% | 94.40% |
| 52314392 | 67.40% | 94.50% |
| 36136854 | 67.80% | 94.70% |
| 53585938 | 69.30% | 94.30% |
| 51826599 | 70.00% | 94.30% |
| 33160465 | 69.80% | 94.50% |
| 35367885 | 70.00% | 94.40% |
| 32006636 | 68.30% | 94.40% |
| 36408961 | 72.10% | 93.70% |
| 34450397 | 69.80% | 94.20% |
| 25640813 | 63.50% | 94.60% |
| 35536282 | 71.10% | 93.80% |
| 39838718 | 74.40% | 93.80% |
| 55376274 | 72.00% | 94.60% |
| 35157235 | 69.90% | 94.60% |
| 33716613 | 68.10% | 94.60% |
| 72072972 | 71.40% | 95.40% |
| 38939420 | 68.90% | 94.90% |
| 32687659 | 69.60% | 94.30% |
| 34027480 | 67.60% | 94.90% |
| 41466171 | 69.30% | 95.00% |
| 33624275 | 69.30% | 94.40% |
| 34669601 | 69.30% | 94.40% |
| 61262472 | 64.80% | 97.60% |
| 36576680 | 73.70% | 93.80% |
| 53767295 | 66.70% | 95.20% |
| 47013530 | 70.90% | 95.00% |
| 35042637 | 68.80% | 94.10% |
| 31827470 | 69.00% | 94.30% |
| 33817465 | 68.30% | 94.50% |
| 30241420 | 69.80% | 94.50% |
| 37665803 | 70.40% | 94.50% |
| 29032832 | 67.60% | 94.80% |
| 32114865 | 69.10% | 94.60% |
| 33744199 | 69.40% | 94.40% |
| 34474753 | 69.80% | 94.70% |
| 35033123 | 69.60% | 94.90% |

| | | |
|---|---|---|
| 33373283 | 68.90% | 94.60% |
| 55438623 | 68.90% | 95.10% |
| 44115659 | 69.50% | 94.10% |
| 30780094 | 66.70% | 95.00% |
| 34107321 | 70.30% | 94.60% |
| 31876178 | 68.00% | 94.70% |
| 40099907 | 69.80% | 94.70% |
| 34831867 | 67.00% | 94.70% |
| 34627876 | 68.80% | 94.60% |
| 95497833 | 72.40% | 97.80% |
| 31684332 | 67.80% | 94.50% |
| 31390535 | 69.20% | 94.30% |
| 30613838 | 71.20% | 93.90% |
| 30990271 | 67.10% | 94.80% |
| 30118220 | 71.60% | 94.00% |
| 33112214 | 67.90% | 94.60% |
| 33035836 | 65.50% | 95.00% |
| 35537586 | 69.70% | 94.40% |
| 29194220 | 69.40% | 94.10% |
| 37114963 | 73.00% | 93.90% |
| 35821160 | 70.80% | 94.10% |
| 41939328 | 69.50% | 94.60% |
| 34338754 | 63.60% | 94.80% |
| 39344056 | 66.40% | 94.40% |
| 42388855 | 65.30% | 94.70% |
| 50778466 | 73.60% | 94.60% |
| 34985081 | 71.90% | 94.40% |
| 28569972 | 68.50% | 94.60% |
| 32898431 | 72.50% | 94.40% |
| 57051860 | 73.00% | 94.60% |
| 42514381 | 68.60% | 94.90% |
| 30691984 | 67.80% | 94.10% |
| 30004029 | 71.20% | 93.70% |
| 37151771 | 74.20% | 94.30% |

| Number_of_on_target_genotypes_more_than_1X | Coverage_of_target_regions_more_than_10X |
|---|---|
| 58198378 | 83.90% |
| 58413120 | 84.90% |
| 58792872 | 86.00% |
| 58505981 | 88.30% |
| 60587563 | 91.70% |
| 58418725 | 82.90% |
| 58668633 | 87.20% |
| 58583939 | 85.00% |
| 58686853 | 88.40% |
| 58778533 | 87.70% |
| 58559587 | 88.80% |
| 58567749 | 88.80% |
| 58641503 | 85.80% |
| 58625976 | 85.90% |
| 58601592 | 85.10% |
| 58194066 | 86.90% |
| 58492663 | 86.20% |
| 58744210 | 83.30% |
| 58249831 | 86.90% |
| 58256969 | 85.60% |
| 58760103 | 89.00% |
| 58732834 | 86.60% |
| 58739602 | 86.00% |
| 59234020 | 91.90% |
| 58909865 | 87.40% |
| 58519214 | 85.00% |
| 58904569 | 86.70% |
| 58955347 | 87.50% |
| 58620139 | 86.50% |
| 58605537 | 86.00% |
| 60572499 | 92.60% |
| 58227825 | 85.40% |
| 59076695 | 89.10% |
| 58968028 | 89.30% |
| 58446590 | 85.70% |
| 58515954 | 84.70% |
| 58663173 | 86.10% |
| 58642622 | 86.40% |
| 58691213 | 87.00% |
| 58839935 | 84.50% |
| 58746433 | 85.80% |
| 58594201 | 86.00% |
| 58765828 | 87.10% |
| 58918881 | 87.30% |

| | |
|---|---|
| 58753045 | 86.00% |
| 59055136 | 89.80% |
| 58419000 | 87.20% |
| 59012015 | 85.60% |
| 58761357 | 86.20% |
| 58810588 | 85.60% |
| 58811244 | 87.60% |
| 58807862 | 85.90% |
| 58736130 | 86.20% |
| 60739184 | 94.70% |
| 58656961 | 85.20% |
| 58562734 | 85.10% |
| 58321714 | 85.90% |
| 58886058 | 84.80% |
| 58361200 | 84.40% |
| 58710989 | 85.40% |
| 58971278 | 86.20% |
| 58630592 | 86.50% |
| 58431128 | 84.90% |
| 58312074 | 85.90% |
| 58415332 | 86.20% |
| 58722461 | 87.50% |
| 58850959 | 86.70% |
| 58584520 | 86.70% |
| 58790884 | 87.30% |
| 58712432 | 88.70% |
| 58609894 | 85.70% |
| 58748097 | 84.90% |
| 58634698 | 87.10% |
| 58713018 | 88.80% |
| 58919725 | 88.40% |
| 58442553 | 82.70% |
| 58180252 | 82.50% |
| 58526066 | 87.90% |

| Number_of_on_target_genotypes_more_than_10X | Mean_depth_of_target_regions | Number_of_SNPs |
|---|---|---|
| 52061681 | 47.1 | 67055 |
| 52703299 | 44.9 | 70909 |
| 53368582 | 43.4 | 73319 |
| 54827338 | 59 | 71346 |
| 56925801 | 77.9 | 90457 |
| 51468425 | 33.6 | 79632 |
| 54148389 | 52.3 | 78017 |
| 52774095 | 42.1 | 71657 |
| 54906530 | 67.1 | 69194 |
| 54460204 | 47.4 | 73204 |
| 55112637 | 68.9 | 72483 |
| 55147605 | 66.7 | 75002 |
| 53277630 | 42.7 | 71793 |
| 53347417 | 45.3 | 71276 |
| 52826315 | 42.1 | 70166 |
| 53939564 | 47.5 | 72804 |
| 53545811 | 45.2 | 71591 |
| 51739782 | 33.3 | 71030 |
| 53958549 | 46.5 | 72716 |
| 53143538 | 51.3 | 67274 |
| 55249168 | 71.2 | 76284 |
| 53740596 | 45.3 | 71756 |
| 53408516 | 43.6 | 72619 |
| 57029153 | 93.8 | 77913 |
| 54249074 | 51.2 | 87164 |
| 52783396 | 42.3 | 67522 |
| 53836101 | 44.8 | 83655 |
| 54345688 | 54.5 | 89498 |
| 53710150 | 43.3 | 72555 |
| 53385200 | 44.8 | 72053 |
| 57466609 | 80.8 | 90825 |
| 53003054 | 46.9 | 71532 |
| 55340813 | 68.3 | 73982 |
| 55444978 | 60 | 82059 |
| 53186979 | 45.6 | 72199 |
| 52605303 | 41.2 | 72086 |
| 53476868 | 43.8 | 72840 |
| 53613701 | 38.9 | 72522 |
| 54014596 | 48.8 | 71484 |
| 52431359 | 37.9 | 65435 |
| 53269418 | 41.8 | 73532 |
| 53419634 | 43.9 | 73528 |
| 54091652 | 44.2 | 73079 |
| 54170210 | 44.9 | 73727 |

| | | |
|---|---|---|
| 53423532 | 43 | 72851 |
| 55726405 | 71.5 | 76841 |
| 54136504 | 57.4 | 74019 |
| 53118680 | 39.6 | 71569 |
| 53504049 | 43.7 | 73627 |
| 53136276 | 41 | 71671 |
| 54390499 | 51.4 | 73890 |
| 53323601 | 44.8 | 72705 |
| 53507652 | 44.3 | 72712 |
| 58790009 | 126.9 | 83956 |
| 52873317 | 40.6 | 71085 |
| 52833652 | 40.2 | 72889 |
| 53343554 | 39.8 | 72484 |
| 52671202 | 40.1 | 72041 |
| 52427879 | 38.6 | 71676 |
| 53042383 | 42.8 | 72616 |
| 53497566 | 42.8 | 72730 |
| 53723497 | 46 | 67871 |
| 52689095 | 37.8 | 71959 |
| 53302732 | 47.8 | 71019 |
| 53541866 | 46.4 | 71978 |
| 54349328 | 54.2 | 74719 |
| 53847938 | 44.7 | 72102 |
| 53856624 | 50.5 | 71163 |
| 54225799 | 54.8 | 71978 |
| 55090401 | 65.2 | 75535 |
| 53177640 | 45.1 | 69234 |
| 52714915 | 36.9 | 66329 |
| 54087378 | 42.2 | 75138 |
| 55155683 | 73.8 | 75396 |
| 54892642 | 55 | 74907 |
| 51332080 | 39.5 | 71556 |
| 51210079 | 38.2 | 69921 |
| 54596727 | 47.8 | 74022 |

| Number_of_coding_SNPs | Number_of_synonymous_SNPs | Number_of_nonsynonymous_SNPs |
|---|---|---|
| 18696 | 9643 | 8576 |
| 19738 | 10132 | 9112 |
| 20169 | 10367 | 9266 |
| 19183 | 9854 | 8861 |
| 23967 | 12527 | 10848 |
| 23237 | 12035 | 10630 |
| 21601 | 11290 | 9799 |
| 20089 | 10296 | 9278 |
| 18560 | 9510 | 8536 |
| 19866 | 10186 | 9165 |
| 19363 | 9969 | 8914 |
| 20032 | 10330 | 9207 |
| 19771 | 10186 | 9071 |
| 19600 | 10108 | 8979 |
| 19200 | 9889 | 8810 |
| 19664 | 10263 | 8930 |
| 19537 | 10140 | 8928 |
| 19836 | 10183 | 9169 |
| 19619 | 10075 | 9046 |
| 18605 | 9522 | 8624 |
| 20136 | 10448 | 9213 |
| 19672 | 10148 | 9016 |
| 19967 | 10249 | 9213 |
| 20020 | 10478 | 9057 |
| 24476 | 12779 | 11103 |
| 18739 | 9730 | 8518 |
| 23758 | 12271 | 10907 |
| 25066 | 13020 | 11436 |
| 19975 | 10345 | 9143 |
| 19892 | 10231 | 9127 |
| 23882 | 12376 | 10883 |
| 19861 | 10268 | 9059 |
| 19682 | 10284 | 8917 |
| 22617 | 11659 | 10368 |
| 19776 | 10224 | 9049 |
| 20002 | 10296 | 9186 |
| 20002 | 10300 | 9190 |
| 19994 | 10345 | 9122 |
| 19597 | 10090 | 9001 |
| 18188 | 9332 | 8378 |
| 20444 | 10531 | 9393 |
| 20205 | 10409 | 9266 |
| 20179 | 10331 | 9349 |
| 20237 | 10517 | 9189 |

| | | |
|---|---|---|
| 20241 | 10424 | 9264 |
| 20442 | 10646 | 9276 |
| 20167 | 10509 | 9162 |
| 19785 | 10210 | 9060 |
| 20297 | 10455 | 9310 |
| 19959 | 10304 | 9136 |
| 20085 | 10430 | 9155 |
| 20079 | 10340 | 9189 |
| 19891 | 10194 | 9173 |
| 20551 | 10733 | 9296 |
| 19655 | 10189 | 8974 |
| 20183 | 10374 | 9287 |
| 20089 | 10387 | 9213 |
| 19875 | 10209 | 9157 |
| 20112 | 10457 | 9187 |
| 19940 | 10347 | 9074 |
| 20040 | 10298 | 9201 |
| 18447 | 9576 | 8395 |
| 19901 | 10238 | 9146 |
| 19578 | 10105 | 8965 |
| 19805 | 10243 | 9066 |
| 20084 | 10425 | 9172 |
| 19559 | 10087 | 8953 |
| 19310 | 9924 | 8897 |
| 19394 | 10002 | 8886 |
| 20390 | 10522 | 9319 |
| 18939 | 9711 | 8736 |
| 18244 | 9292 | 8449 |
| 20817 | 10725 | 9547 |
| 20251 | 10410 | 9339 |
| 20187 | 10479 | 9217 |
| 20063 | 10283 | 9288 |
| 19888 | 10234 | 9160 |
| 20501 | 10530 | 9459 |

| Number_of_indels | Number_of_coding_indels |
| --- | --- |
| 6847 | 342 |
| 6928 | 348 |
| 7442 | 351 |
| 7885 | 377 |
| 9552 | 532 |
| 7937 | 434 |
| 8156 | 415 |
| 7142 | 365 |
| 7742 | 376 |
| 7942 | 371 |
| 7800 | 363 |
| 8081 | 364 |
| 7273 | 361 |
| 7114 | 386 |
| 7604 | 348 |
| 7753 | 351 |
| 7661 | 381 |
| 7498 | 353 |
| 7747 | 357 |
| 7085 | 351 |
| 7983 | 364 |
| 7373 | 367 |
| 7383 | 360 |
| 8801 | 407 |
| 8824 | 445 |
| 7189 | 344 |
| 8469 | 411 |
| 8958 | 445 |
| 7339 | 344 |
| 7333 | 365 |
| 10225 | 595 |
| 7071 | 369 |
| 7863 | 384 |
| 8222 | 423 |
| 7332 | 337 |
| 7336 | 368 |
| 7306 | 359 |
| 7348 | 382 |
| 7461 | 378 |
| 7034 | 343 |
| 7443 | 390 |
| 7476 | 373 |
| 7275 | 375 |
| 7347 | 375 |

| | |
|---|---|
| 7243 | 377 |
| 7680 | 380 |
| 7375 | 358 |
| 7070 | 368 |
| 7148 | 379 |
| 7142 | 348 |
| 7479 | 361 |
| 7287 | 374 |
| 7241 | 370 |
| 9218 | 464 |
| 7046 | 365 |
| 7185 | 354 |
| 7505 | 370 |
| 7350 | 355 |
| 7005 | 355 |
| 7237 | 342 |
| 7323 | 361 |
| 7208 | 353 |
| 7266 | 367 |
| 7037 | 371 |
| 7329 | 361 |
| 7574 | 368 |
| 7666 | 361 |
| 7641 | 369 |
| 7711 | 355 |
| 7737 | 405 |
| 7255 | 350 |
| 7087 | 339 |
| 7735 | 402 |
| 7854 | 375 |
| 7888 | 370 |
| 7084 | 331 |
| 6744 | 326 |
| 7415 | 379 |
