# Supplementary material for: Integrative analysis of mutational and transcriptional profiles reveals driver mutations of metastatic breast cancers
Source: Cell Discov. 2016 Aug 30;2:16025–. doi: 10.1038/celldisc.2016.25 (PMC5004232; doi:10.1038/celldisc.2016.25)
Supplement: Supplementary Table S3 [file celldisc201625-s8.pdf]

Supplementary Table 3. 53 HRM-specific mutations

Annotation	Mutation Type	Nucleotide Number	cDNA mutation	Impact on Protein Synthesis
ADPGK	Non-synonymous SNV	NM_031284	C316T	H106Y
ARMC2	Non-synonymous SNV	NM_032131	C455A	P152H
C11orf40	Frameshift Deletion	NM_144663	341_342del	114_114del
C3orf14	Stop-gain SNV	NM_020685	C382T	R128X
C6orf132	Non-frameshift Deletion	NM_001164446	2922_2924del	974_975del
CDC27	Stop-gain SNV	NM_001114091	C710A	S237X
CELA3B	Non-synonymous SNV	NM_007352	T800A	I267K
CENPL	Non-synonymous SNV	NM_033319	T484G	F162V
CHTOP	Non-synonymous SNV	NM_015607	C427G	R143G
CLSPN	Non-frameshift Deletion	NM_022111	3583_3585del	1195_1195del
COL5A3	Non-synonymous SNV	NM_015719	A4750C	T1584P
CPOX	Non-synonymous SNV	NM_000097	T1174G	Y392D
DLL4	Non-synonymous SNV	NM_019074	G1699T	A567S
DYRK1B	Non-synonymous SNV	NM_004714	A1823C	D608A
EIF2C4	Non-synonymous SNV	NM_017629	A1838C	H613P
EMILIN3	Non-synonymous SNV	NM_052846	T1952G	V651G
FGFRL1	Non-synonymous SNV	NM_001004356	T119G	V40G
FRG2B	Non-synonymous SNV	NM_001080998	G300C	M100I
GOLGA6L10	Non-synonymous SNV	NM_001164465	G1025A	R342Q
HAX1	Non-frameshift Deletion	NM_006118	119_121del	40_41del
HBP1	Frameshift Insertion	NM_012257	534_535ins	H178fs
KDM6B	Non-frameshift Deletion	NM_001080424	786_791del	262_264del
KLHL6	Non-synonymous SNV	NM_130446	A1390C	T464P
LIMK1	Non-synonymous SNV	NM_002314	A1841C	H614P
MUC6	Non-synonymous SNV	NM_005961	A3211C	T1071P
NUP93	Stop-gain SNV	NM_014669	C43T	Q15X
OR13A1	Frameshift Insertion	NM_001004297	805_806ins	Y269fs
PCGF6	Frameshift Deletion	NM_001011663	68_69del	23_23del
PGM3	Non-synonymous SNV	NM_001199917	G552T	L184F
PKP2	Non-synonymous SNV	NM_001005242	A2171C	K724T
PKP3	Non-synonymous SNV	NM_007183	G830C	R277P
POTEH	Non-synonymous SNV	NM_001136213	C324G	C108W
PRAMEF10	Non-synonymous SNV	NM_001039361	T503G	F168C
PRAMEF11	Non-synonymous SNV	NM_001146344	A646T	I216F
PRAMEF11	Stop-gain SNV	NM_001146344	T498A	C166X
PRAMEF16	Non-synonymous SNV	NM_001045480	A923G	Q308R
PRB3	Non-synonymous SNV	NM_006249	A703C	K235Q
PRR25	Non-synonymous SNV	NM_001013638	C720A	H240Q

PSMD2	Non-synonymous SNV	NM_002808	A515G	E172G
RTEL1	Non-synonymous SNV	NM_016434	G901A	A301T
SATL1	Stop-gain SNV	NM_001012980	C1803G	Y601X
SEPT8	Non-synonymous SNV	NM_001098812	T1300G	S434A
SHROOM4	Non-Frameshift Insertion	NM_020717	3392_3393ins	Q1131delinsQE
SLC18A3	Non-synonymous SNV	NM_003055	A241C	T81P
SLC22A5	Non-synonymous SNV	NM_003060	G259C	A87P
SRP14	Non-frameshift Deletion	NM_003134	321_323del	107_108del
TAF4	Non-synonymous SNV	NM_003185	A2385C	K795N
TAS1R2	Non-synonymous SNV	NM_152232	A1885C	T629P
TAS2R30	Non-synonymous SNV	NM_001097643	A521T	H174L
TIE1	Non-synonymous SNV	NM_005424	T14G	V5G
WDR66	Non-frameshift Deletion	NM_144668	185_187del	62_63del
ZNF295	Non-frameshift Deletion	NM_001098402	2649_2651del	883_884del
ZNF492	Non-synonymous SNV	NM_020855	G118A	A40T
