# Supplementary material for: Integrative analysis of mutational and transcriptional profiles reveals driver mutations of metastatic breast cancers
Source: Cell Discov. 2016 Aug 30;2:16025–. doi: 10.1038/celldisc.2016.25 (PMC5004232; doi:10.1038/celldisc.2016.25)
Supplement: Supplementary Table S4 [file celldisc201625-s9.pdf]

Supplementary Table 4. HRM-specific TFs

TF	p-value
PAX5	0.000851
FOS	0.004978
SREBF1	0.000851
MYB	0.000652
TCF7	0.006019
FLI1	0.000273
SP1	0.009989
IRF1	0.000301
AKR1B1	0.002507
ELK1	0.008377
IRF8	0.006019
JUN	0.000273
USF2	0.000851
CEBPA	3.38E-13
SPI1	3.24E-17
PPARG	1.40E-05
IKZF1	0.000552
E2F6	2.72E-06
ETS1	3.39E-13
POU2F2	7.19E-07
