# Supplementary material for: Integrative analysis of mutational and transcriptional profiles reveals driver mutations of metastatic breast cancers
Source: Cell Discov. 2016 Aug 30;2:16025–. doi: 10.1038/celldisc.2016.25 (PMC5004232; doi:10.1038/celldisc.2016.25)
Supplement: Supplementary Table S5 [file celldisc201625-s10.pdf]

Supplementary Table 5. 13 mutant genes whose pathways were detected

Rank	Mutant gene	Target TF	Detected by mathematic model?
1	ADPGK	PAX5	
2	SLC22A5	PPARG	
3	PCGF6	E2F6	Yes
4	DYRK1B	SP1	
5	NUP93	E2F6	Yes
6	PKP2	FOS	Yes
7	LIMK1	PPARG	Yes
8	TIE1	FOS	Yes
9	CDC27	FOS	Yes
10	DLL4	SPI1	Yes
11	HAX1	SP1	
12	SEPT8	PPARG	
13	KLHL6	SP1	
