# Supplementary material for: Integrative analysis of mutational and transcriptional profiles reveals driver mutations of metastatic breast cancers
Source: Cell Discov. 2016 Aug 30;2:16025–. doi: 10.1038/celldisc.2016.25 (PMC5004232; doi:10.1038/celldisc.2016.25)
Supplement: Supplementary Table S6 [file celldisc201625-s11.pdf]

Supplementary Table 6. HRM-specific pathways linking mutations to TFs

For each mutation, the pathway shown here is the shortest pathway linking the mutation to associated TFs.

Mutation	TF	Pathway components
LIMK1	PPARG	LIMK1, HSP90AA1, CAV1, PPARG
HAX1	SP1	HAX1, ESR1, SP1
SEPT8	PPARG	SEPT8, SEPT5, STX4, PPARG
ADPGK	PAX5	ADPGK, TADA3, TP53, CR2, PAX5
DYRK1B	SP1	DYRK1B, CCND1, SP1
NUP93	E2F6	NUP93, RAE1, BUB1, CDK1, E2F1, E2F6
KLHL6	SP1	KLHL6, PPP1R1B, CSNK2A1, SP1
DLL4	SPI1	DLL4, NOTCH1, SPI1
PCGF6	E2F6	PCGF6, E2F6
CDC27	FOS	CDC27, SMAD3, FOS
SLC22A5	PPARG	SLC22A5, SLC9A3R1, EGFR, CAV1, PPARG
TIE1	FOS	TIE1, SMAD3, FOS
PKP2	FOS	PKP2, MARK3, MITF, FOS
